# Morphology effect of a novel biocompatible nucleic acid delivery nanosystem of g‐C_3_N_4_@dsRNA for application in plant gene expression and plant virus disease protection

**DOI:** 10.1111/pbi.70189

**Published:** 2025-06-18

**Authors:** Xuefeng Wei, Guangjin Fan, Song Yang, Xianchao Sun, Lin Cai

**Affiliations:** ^1^ State Key Laboratory of Green Pesticides; Key Laboratory of Green Pesticide and Agricultural Bioengineering, Ministry of Education Center for R&D of Fine Chemicals of Guizhou University Guiyang China; ^2^ Guizhou Provincial Key Laboratory for Tobacco Quality Improvement and Efficiency Enhancement College of Tobacco Science of Guizhou University Guiyang China; ^3^ College of Plant Protection Southwest University Chongqing China

**Keywords:** nucleic acid delivery nanosystem, g‐C_3_N_4_, TMV, morphology effect

## Abstract

Delivery of exogenous nucleic acid to intact plants is a desirable but challenging technology due to the dominant transport barrier posed by the plant cell wall. Here, we found that three different morphologies of g‐C_3_N_4_ nanomaterials synthesized from urea can assist in the delivery of exogenous nucleic acids into mature leaf cells of *Nicotiana benthamiana*. Among these, g‐C_3_N_4_ carbon dots (CDs) showed a higher ability to deliver exogenous nucleic acid compared to nanoporous and g‐C_3_N_4_ nanosheets. The delivery ability of exogenous dsRNA and plasmid DNA by g‐C_3_N_4_ could also be restrained by stomatal closure and the endocytosis pathways in plant cells. Furthermore, the coupling of g‐C_3_N_4_ CDs with dsCP (CDs@dsCP), which is the dsRNA matching a specific fragment of the *Coat protein*‐encoding gene of TMV, resulted in a superior antiviral effect compared with other morphologies of g‐C_3_N_4_@dsRNAs and other loaded dsRNAs, which match *Replicase*, *RNA‐dependent Replicase* or *Movement protein* of TMV. Significantly, a single spray of CDs@dsCP provided virus protection for at least 5 days. In addition, the g‐C_3_N_4_ CDs and g‐C_3_N_4_ CDs@dsRNA had no adverse effects on plant growth and development. Overall, our study presents a novel biocompatible and convenient tool for gene expression or gene silencing in intact plant leaves by spraying g‐C_3_N_4_ nanomaterials encapsulated with DNA or dsRNA, the efficiency of which is affected by the morphology of the g‐C_3_N_4_ nanomaterial, stomatal state and plant endocytosis pathway, and a highly promising solution for plant virus disease, which is an unsolved problem in plant disease control.

## Introduction

In agriculture, genetic enhancement of plants can be employed to create crops that give higher yields and are resistant to diseases, insects and abiotic stress (Wang *et al*., 2022; War *et al*., 2012; Zhang *et al*., 2022b). Despite several decades of advances in plant biotechnology, most plant species remain difficult to transform genetically (Anjanappa and Gruissem, 2021). The bottlenecks hindering the successful delivery of exogenous nucleic acids into mature plants include: (1) biomolecule delivery barriers imposed by the rigid multilayered cell wall, (2) narrow host range of Agrobacterium strains and (3) limited utilization of plant cell totipotency (Anjanappa and Gruissem, 2021). Traditional delivery methods for artificial exogenous nucleic acids primarily rely on biological vectors (Agrobacterium‐mediated, viral vectors), physical approaches (biolistic particle delivery) and protoplast‐based systems (PEG‐mediated, electroporation and microinjection). Compared to permanent genetic modification through transgenic approaches, trait engineering via transient expression of exogenous nucleic acids (without genome integration) may demonstrate improved regulatory compliance (Wang *et al*., 2019). All previous traditional artificial exogenous nucleic acids delivery methods are difficult to implement conveniently. However, unlike well‐established biomolecule delivery systems in animal cells, achieving efficient nucleic acid delivery in mature plants with intact cell walls remains scientifically challenging, principally due to low transmembrane transport efficiency through plant cell walls (Wang *et al*., 2016b, 2019).

In recent years, a small number of studies have successfully achieved cytoplasmic delivery of exogenous nucleic acid into intact plant cells for gene expression, gene silencing and even gene editing without the need for external forces by using different nanomaterial‐assisted delivery systems, including clay nanosheets, carbon nanotubes, layered double hydroxide nanoparticles, graphene oxide nanoparticles, mesoporous silica nanoparticles, gold nanoparticles, star polycations and liposome nanoparticles (Cai *et al*., 2024; Demirer *et al*., 2020; Hao *et al*., 2024; Li *et al*., 2022; Mitter *et al*., 2017; Wang *et al*., 2023; Zhang *et al*., 2022a). These delivery nanosystems have the following advantages: (1) nanocarriers can protect RNAs from enzymatic degradation after application to plants; (2) nanocarriers enable controlled delivery of nucleic acids to plant cells, including sustained release and long‐term retention, subcellular organ‐targeted delivery and stimulated release; and (3) the nanoparticle‐mediated delivery system is highly versatile both with regard to applicability to all plant species and the capacity to deliver different forms of nucleic acids (Yong *et al*., 2024). Nanomaterial‐based delivery systems are being established to address the limitations of the current methods for the introduction of exogenous genetic material into plants and are proving to be a feasible, versatile and efficient approach for facilitating the incorporation of functional RNA and DNA. Nevertheless, there remains a critical need to develop additional nanocarriers capable of efficiently delivering nucleic acids into plants, thereby expanding the currently limited repertoire of delivery systems. In particular, nano‐delivery systems with high delivery efficiency, no external mechanical pressure, good biocompatibility, nontoxicity, degradability, and versatility are ideal for plant scientists. Recent studies have shown that multiple delivery nanosystems constitute an ideal feasible approach to circumvent dsRNA degradation, traverse plant cell barriers and evade potential immune system recognition (Hou *et al*., 2021; Wang *et al*., 2016b, 2019; Zhang *et al*., 2021). However, there is a lack of knowledge regarding the effects of different morphologies and structures of the same type of nano‐delivery material on the delivery ability of exogenous nucleic acid.

Graphitic carbon nitride (g‐C_3_N_4_) is a unique layered and controllable carbon‐based nanomaterial that is easy to manufacture, cost‐effective, environmentally friendly, has abundant electrons and has been mainly used in the fields of photocatalysis, electrocatalysis and heterogeneous catalysis. g‐C_3_N_4_ has been shown to be more effective than other nonmetal‐based nanomaterials in wastewater heavy metal detection and purification (Wang and Wang, 2022; Zhu *et al*., 2014). g‐C_3_N_4_ is also being used to develop highly sensitive fluorescent detection systems for environmental pollutants (Qu *et al*., 2022). In a study of the effects of g‐C_3_N_4_ on plants, g‐C_3_N_4_ was not only found to be non‐toxic to plants but could also alleviate cadmium‐ and arsenate‐induced phytotoxicity in rice (Hao *et al*., 2023). In addition, our previous studies revealed that g‐C_3_N_4_ has concentration‐dependent and light‐dependent anti‐phytopathogen effects on *Pseudomonas syringae* and *Phytophthora capsici*, which are two different types of important plant pathogens (Cai *et al*., 2021a,b). Recently, direct heat treatment has enabled the large‐scale synthesis of g‐C_3_N_4_ by precisely controlling the thermal condensation temperature of a low‐cost urea precursor (Fang *et al*., 2016). The g‐C_3_N_4_‐based material has high potential to meet the safety requirements for exogenous nucleic acid delivery into plant cells because urea or melamine, which are its sole synthetic precursors, are highly biocompatible and metabolizable in plants (Liao *et al*., 2020; Wang *et al*., 2011; Zhang *et al*., 2013b). Thus, we hypothesize that g‐C_3_N_4_ may be a suitable material for delivering exogenous nucleic acids into intact plant cells owing to its controllable structure, non‐toxicity, low cost, high biocompatibility and abundant electrons.

To further verify our hypothesis about the potential of g‐C_3_N_4_ for delivering exogenous nucleic acids into plant cells and investigate the effect of g‐C_3_N_4_ morphology on its ability to deliver exogenous nucleic acids, three different morphologies of g‐C_3_N_4_, including carbon dots (CDs), nanoporous and nanosheets, were synthesized by using urea or melamine as the sole precursor. These different shapes of g‐C_3_N_4_ were modified with polyethylenimine (PEI) and successfully loaded with dsRNA or plasmid DNA. The g‐C_3_N_4_ CDs showed higher nucleic acid delivery capability than g‐C_3_N_4_ nanoporous and nanosheets forms. Furthermore, these three different shapes of g‐C_3_N_4_‐derived dsRNA delivery exhibited pore‐ and endocytosis‐dependent properties. Finally, the nucleic acid delivery system of g‐C_3_N_4_ CDs@dsRNA can achieve long‐lasting protective effects on plants against viral diseases by delivering dsRNA to target key genes of TMV by spraying, which is a safe method for promoting plant health.

## Results

### Synthesis and characterization of three different shapes of g‐C_3_N_4_ that can be coupled with dsRNA

**Figure 1:**
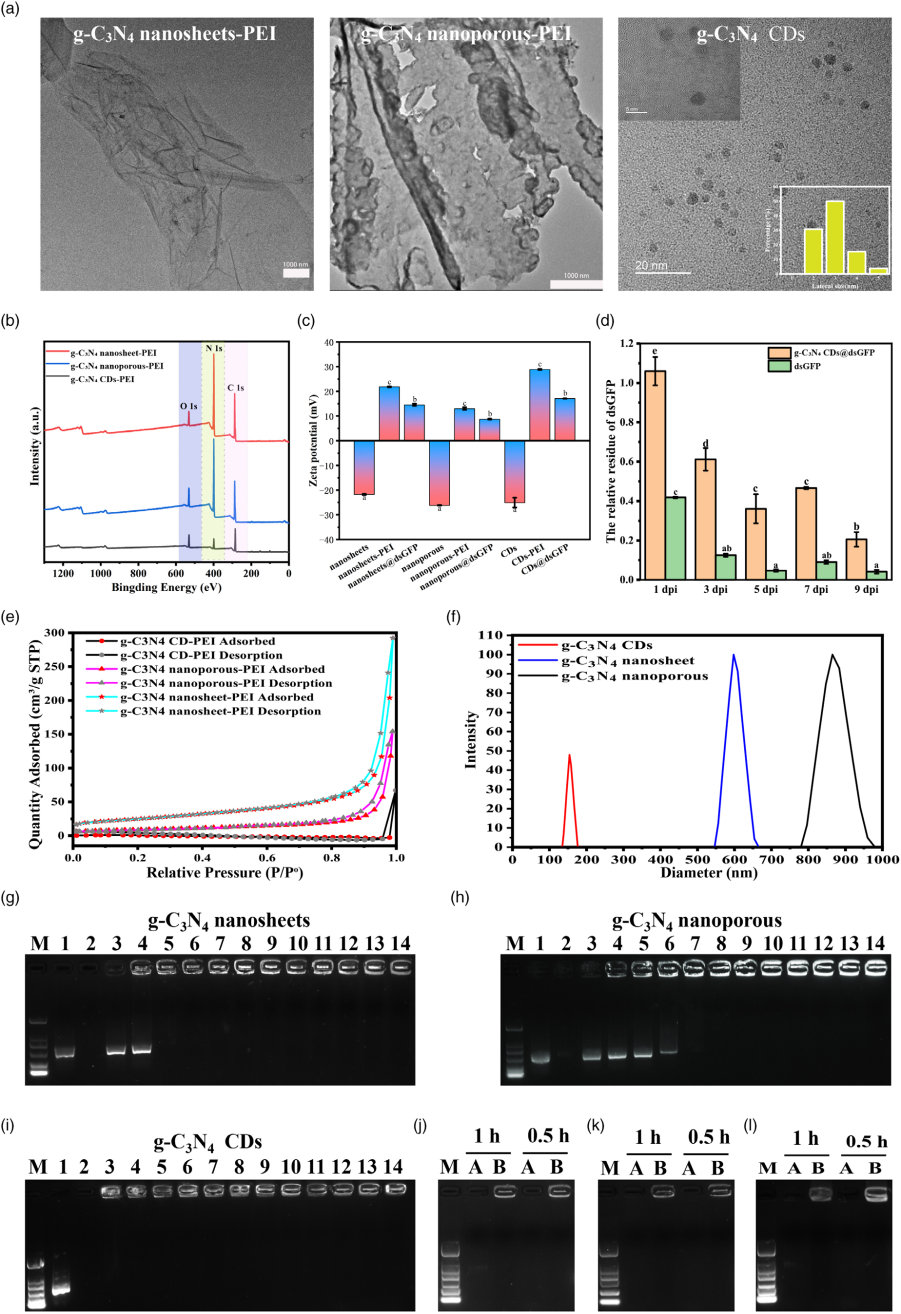
Synthesis and characterization of nanocomposites comprising g‐C_3_N_4_@dsRNA with diverse morphologies. (a) TEM images of g‐C_3_N_4_ nanosheet‐PEI (scale bar, 1000 nm), g‐C_3_N_4_ nanoporous‐PEI (scale bar, 1000 nm) and g‐C_3_N_4_ CDs‐PEI (scale bar, 20 nm). The inset in the top‐left corner of CDs shows the high‐resolution lattice fringes, and the bottom‐right inset shows the size distribution histogram of CDs, obtained by statistical analysis of 30 particles. (b) XPS of g‐C_3_N_4_ nanosheet‐PEI, g‐C_3_N_4_ nanoporous‐PEI and g‐C_3_N_4_ CDs‐PEI. (c) Zeta potential analysis of the g‐C_3_N_4_ nanosheet, g‐C_3_N_4_ nanosheet‐PEI, g‐C_3_N_4_ nanosheet@dsGFP, g‐C_3_N_4_ nanoporous, g‐C_3_N_4_ nanoporous‐PEI, g‐C_3_N_4_ nanoporous@dsGFP, g‐C_3_N_4_ CDs, g‐C_3_N_4_ CDs‐PEI and g‐C_3_N_4_ CDs@dsGFP. (d) The qRT‐PCR analysis revealed the relative content of dsGFP when loaded or not loaded onto g‐C_3_N_4_ CDs after spraying on *N. benthamiana* leaves at 1, 3, 5, 7 or 9 days post spray dps (dps). (e) Nitrogen sorption isotherms of g‐C_3_N_4_ CDs, g‐C_3_N_4_ nanoporous and g‐C_3_N_4_ nanosheet. (f) Dynamic light scattering analysis of g‐C_3_N_4_ CDs, g‐C_3_N_4_ nanoporous and g‐C_3_N_4_ nanosheet. (g–i) The presence of dsGFP was detected by employing agarose electrophoresis analysis after combining with g‐C_3_N_4_ at different mass ratios. The sample in lane M is a DNA marker. The sample in lane 1 is free dsGFP. The sample in lane 2 is a nanomaterial that is not loaded with dsRNA, such as g‐C_3_N_4_ nanosheet, nanoporous and CDs. The samples in lanes 3–14 were obtained by combining g‐C_3_N_4_ and dsRNA at mass ratios of 1:10 (lane 3), 1:2 (lane 4), 1:1 (lane 5), 2:1 (lane 6), 3:1 (lane 7), 4:1 (lane 8), 5:1 (lane 9), 6:1 (lane 10), 7:1 (lane 11), 8:1 (lane 12), 9:1 (lane 13) and 10:1 (lane 14), respectively. (j–l) The protective effect of g‐C_3_N_4_ nanosheet (j), g‐C_3_N_4_ nanoporous (k) and g‐C_3_N_4_ CDs (l) on dsRNA was detected by gel electrophoresis. These three g‐C_3_N_4_ nanomaterials were loaded with dsGFP at a 3:1 ratio to generate nanocomposites. Nanocomposites were incubated with 2 U of RNase for 60 or 30 min, followed by electrophoresis. The lane A indicates the free dsGFP, while lane B indicates the nanocomposites of g‐C_3_N_4_@dsRNA.

Three g‐C_3_N_4_ materials with different morphologies, including nanosheets, nanoporous and CDs, were synthesized from urea or melamine as the single precursor according to the previously described methods (Cai et al., 2021b; Zhang et al., 2013a; Zhou et al., 2013). Our TEM revealed that nanosheet‐PEI had a typical lamellar structure, nanoporous‐PEI had many pores on the lamellar structure, and CDs‐PEI had a spheroidal carbon dot with a diameter of approximately 2–4 nm (Figure 1a). As observed in the XPS survey spectra, g‐C_3_N_4_ nanosheet‐PEI, g‐C_3_N_4_ CDs‐PEI and g‐C_3_N_4_ nanoporous‐PEI were mainly composed of C, N and O (Figure 1b). As previously reported, unoxidized g‐C_3_N_4_ hardly exhibited O1s signals, whereas all the as‐synthesized g‐C_3_N_4_ samples presented strong O1s signals, implying that they contained the same oxygen‐containing group (Hong *et al*., 2017). To further elucidate the chemical compositions of the samples, high‐resolution XPS scans of C1s, N1s and O1s of the as‐prepared g‐C_3_N_4_ nanosheet‐PEI, g‐C_3_N_4_ CDs‐PEI and g‐C_3_N_4_ nanoporous‐PEI samples were obtained. As shown in Figure S1a, the C1s XPS spectra contained three peaks located at 284.6, 286.0 and 287.9 eV, corresponding to N–C=N coordination in the framework of g‐C_3_N_4_ and C‐NHX(x = 1,2) on the edges of the heptazine units and adventitious hydrocarbons, respectively (Yu *et al*., 2017). In the N 1 s spectrum, four typical peaks with binding energies of 398.6, 399.8, 400.9 and 404.0 eV were observed, corresponding to the C–N=C groups, amide CONH groups, NHx groups and positive charge localization in the heptazine framework, respectively (Figure S1b) (Li *et al*., 2018; Liang *et al*., 2015). The O 1 s spectra were fitted with four peaks at 533.3, 532.4, 531.8 and 531.2 eV, which corresponded to C–O, C–OH, CONH and C=O, respectively (Li *et al*., 2018) (Figure S1c). Considering that the zeta potentials of the g‐C_3_N_4_ nanosheet, nanoporous and CDs samples were all negative and that the nucleic acids had a negative charge (Figure 1c), these different g‐C_3_N_4_ species were oxidized and functionalized with PEI on their surface, as described in the Materials and Methods. All the PEI‐modified g‐C_3_N_4_ materials had a positive surface charge (Figure 1c), which could easily adsorb with negatively charged nucleic acids. All dsRNA in this study was loaded on the surface of PEI‐modified g‐C_3_N_4_ by electrostatic adsorption, which is similar to some other nucleic acid delivery nanomaterials (Mendes *et al*., 2022; Rai *et al*., 2019). The zeta potential changes of the materials also indicated the accuracy of the synthesis process of g‐C_3_N_4_@dsRNA at each step (Figure 1c). The hydrodynamic diameters of three nanomaterials were determined by DLS, revealing significant size variations: g‐C_3_N_4_ CDs‐PEI showed a particle size distribution of 152.2 ± 12.1 nm, while g‐C_3_N_4_ nanosheet‐PEI and g‐C_3_N_4_ nanoporous‐PEI measured 627.7 ± 9.25 nm and 851.8 ± 12.62 nm, respectively (Figure 1f). Subsequent nitrogen adsorption measurements demonstrated distinct specific surface areas among the three morphologies: 3.36 m^2^/g for g‐C_3_N_4_ CDs‐PEI, 84.42 m^2^/g for g‐C_3_N_4_ nanosheet‐PEI and 31.64 m^2^/g for g‐C_3_N_4_ nanoporous‐PEI (Figure 1e). These results confirmed that g‐C_3_N_4_ nanomaterials with different morphologies loaded with dsRNA were synthesized successfully.

To further assess the binding capacity of functionalized g‐C_3_N_4_ with different morphologies, 400 bp long dsRNAs matching the GFP gene sequence were transcribed in vitro via a transcription kit. Then, dsGFP in different samples was detected by agarose gel electrophoresis after mixing different mass ratios of dsRNA and nanomaterials, whereas dsGFP and g‐C_3_N_4_ were used as controls. According to the principle of agarose electrophoresis, the migration of the complexes of g‐C_3_N_4_@dsGFP differs from that of free dsGFP because the overall charge of the encapsulated dsRNA is different from that of free dsRNA and because the particle size of the complex may be too large for efficient movement. Only dsRNAs that are not bound to g‐C_3_N_4_ can migrate via gel electrophoresis as free dsRNA. According to our agarose gel electrophoresis results, we could deduce that the maximum loading ratio of dsRNA to g‐C_3_N_4_ nanoporous‐PEI was greater than 1:2 (W: W) (Figures 1h and S2b), that of dsRNA to g‐C_3_N_4_ nanosheet‐PEI was greater than 1:1 (W: W) (Figures 1g and S2a) and that of dsRNA to g‐C_3_N_4_ CDs‐PEI even exceeded 10:1 (W: W) (Figures 1i and S2c). Quantitative analysis of agarose gel fluorescence intensity revealed distinct electrophoretic patterns among the three g‐C_3_N_4_@dsRNA complexes, demonstrating their differential binding to dsRNA (Figure S2). These results showed that g‐C_3_N_4_ CDs per unit mass could adsorb more dsRNA than the two other g‐C_3_N_4_ materials.

### Stability of g‐C_3_N_4_ @dsRNA

Previous reports have shown that nanocarriers can effectively protect loaded DNA or RNA from external damage (Cai *et al*., 2024; Demirer *et al*., 2020; Li *et al*., 2022; Mitter *et al*., 2017; Wang *et al*., 2023). To investigate whether dsRNA is protected by g‐C_3_N_4_ against external damage under RNase conditions, g‐C_3_N_4_@dsGFP was treated with RNase A for different periods of time, followed by inactivation with proteinase K, with the same amount of free dsRNA used as the control. The gel electrophoresis results indicated that the dsGFP from nanosheet‐PEI (Figure 1j), g‐C_3_N_4_ nanoporous‐PEI (Figure 1k) and CDs‐PEI (Figure 1l) still could not be completely degraded after incubation with RNase A for even 60 min. These results suggested that the dsRNA loaded into g‐C_3_N_4_‐PEI was stable in the RNase environment. Furthermore, to evaluate the degradation of dsRNA by g‐C_3_N_4_ under natural conditions, a g‐C_3_N_4_@dsGFP dispersion was sprayed on *N. benthamiana* leaves. Full‐length dsGFP on the leaves was detected by RT‐PCR every 2 days. As shown in Figure 1d, more dsGFP remained on the leaves of the CDs@dsGFP treatment group than on those of the free dsGFP treatment group after the same amount of dsGFP was sprayed on the leaves (Figure 1d), indicating that dsRNA was more resistant to degradation in the environment after loading on CDs. The amount of dsGFP remaining on the CDs@dsGFP‐treated leaves at 9 days post spraying (dps) was even five times higher than that of the free dsGFP‐treated group (Figure 1d). All these results showed that dsGFP on g‐C_3_N_4_@dsGFP could be stored for a long time with stronger resistance to degradation after being sprayed on plant leaves.

### Exogenous nucleic acids are successfully facilitated to enter plant cells by g‐C_3_N_4_ nanomaterials with different morphologies

Previous studies have shown that the ability of nanomaterials to penetrate plant cells and tissues depends on the size, morphology and components of the nanomaterial (Valletta *et al*., 2014; Wang *et al*., 2016b; Zhang *et al*., 2022a). In the process of delivering nanomaterials to transport nucleic acids into plant cells, the plant cell peripheral tissues and cell wall present significant barriers. Here, we further tested the ability of g‐C_3_N_4_ nanomaterials with different morphologies to deliver nucleic acids to 16C cells by infiltration and spraying. First, the g‐C_3_N_4_ nanosheet, nanoporous and CDs were labelled with Cy_3_ and the uptake of these three different g‐C_3_N_4_ by plant cells was investigated after the nanomaterials were infiltrated into 16C leaves for 24 h, as described in the Materials and Methods. After the leaves were washed with water, CLSM was used to examine whether the nanomaterials were internalized into the plant cells. As shown in Figure S3, the red fluorescence signal of g‐C_3_N_4_ CDs‐Cy_3_ was clearly observed along the cell contour, whereas the same signal was scarce for g‐C_3_N_4_ nanosheet‐Cy_3_ or g‐C_3_N_4_ nanoporous‐Cy_3_ (Figure S3), implying that g‐C_3_N_4_ CDs have greater potential for entering plant cells. The red fluorescence signal of all three shapes of g‐C_3_N_4_ was observed along the cell contour, indicating that they did not easily enter plant cells directly.

**Figure 2:**
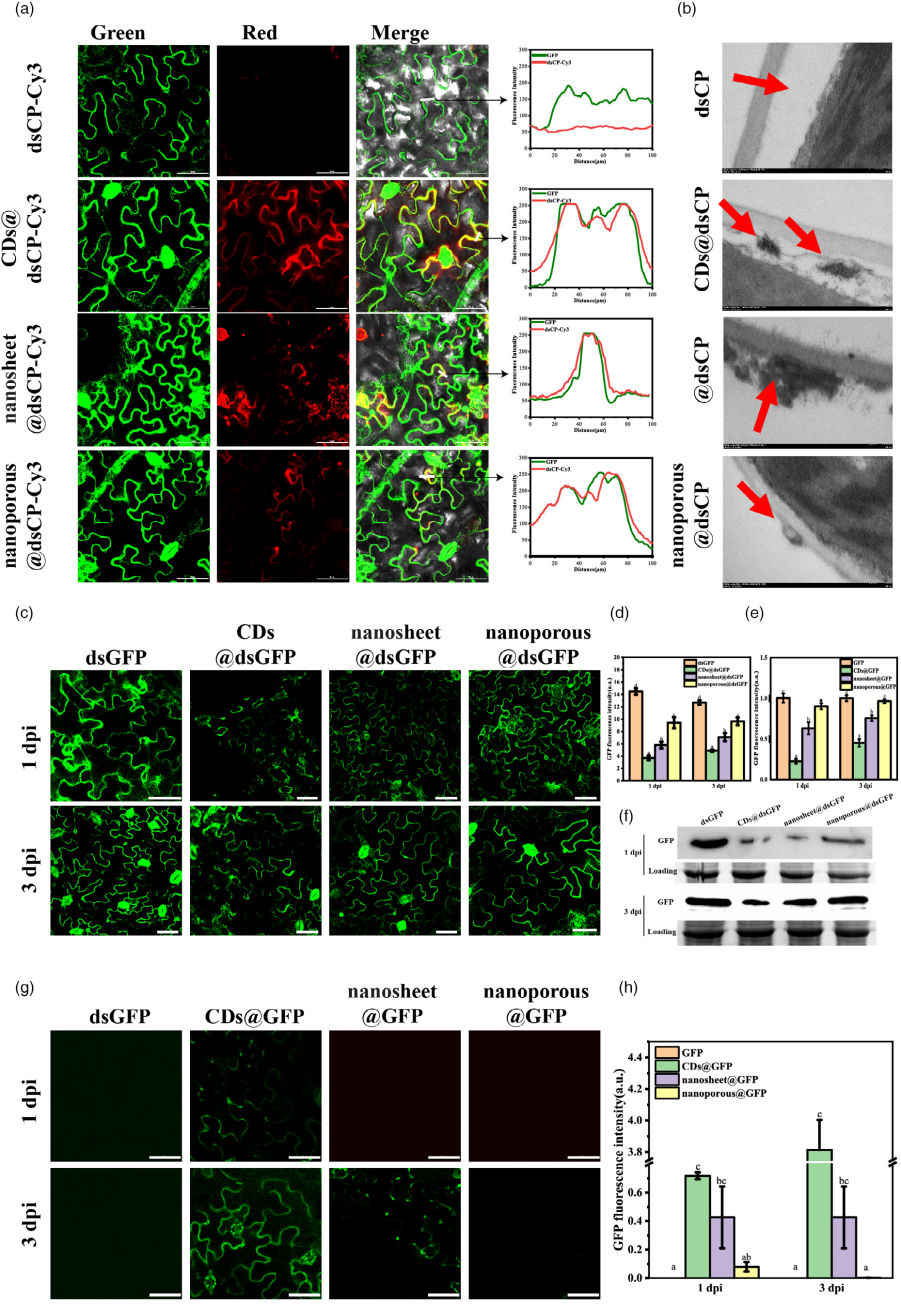
The situation in which exogenous nucleic acids are facilitated by g‐C_3_N_4_ with different morphologies to enter plant cells. (a) Laser confocal photographs showing the subcellular localization of dsCP‐Cy3, g‐C_3_N_4_ CDs@dsCP‐Cy3, g‐C_3_N_4_ nanosheet@dsCP‐Cy3 and g‐C_3_N_4_ nanoporous@dsCP‐Cy3 in leaves of 16C (*N. benthamiana* that expresses GFP consistently) after they were sprayed on 16C leaves for 24 h. The scale bar of all laser confocal is 50 μm. (b) TEM images showing the localization of g‐C_3_N_4_@dsCP at the surrounding of the plant cell wall (red arrow). TEM observation was performed after 24 h infiltration of the nanomaterials into *N. benthamiana* leaves with a headless syringe. No particles were observed near the plant cell wall in the dsCP treatment group, several particles were observed between the cell wall and the cell membrane in the g‐C_3_N_4_ CDs@dsCP group (red arrow), several particles were observed outside the cell wall in the g‐C_3_N_4_ nanosheet@dsCP group (red arrow) and several particles were detected outside the cell wall in the g‐C_3_N_4_ nanoporous@dsCP group (red arrows). (c) Laser confocal photographs showing the GFP silencing of intact plant cells in 16C via g‐C_3_N_4_@dsGFP. The naked dsGFP treatment is the control group. The other three groups, including treatments of g‐C_3_N_4_ CDs@dsGFP, nanosheet@dsGFP and nanoporous@dsGFP, comprised nanomaterials combined with dsGFP at a 4:1 ratio. From left to right, treatments included naked dsGFP, g‐C_3_N_4_‐CDs@dsGFP, g‐C_3_N_4_ nanosheet@dsGFP and g‐C_3_N_4_ nanoporous@dsGFP. The first row depicts confocal microscopy images taken at 1 dps. The second row shows images taken at 3 dps. The scale bar represents 50 μm. (d) Statistical comparison of GFP fluorescence luminance from different treatments, including naked dsGFP, g‐C_3_N_4_ CDs@dsGFP, g‐C_3_N_4_ nanosheet@dsGFP and g‐C_3_N_4_ nanoporous@dsGFP. Each treatment consisted of six leaves, each of which was photographed in five randomly selected locations. Different letters above each bar indicate significant differences at *P* < 0.05 as determined by one‐way ANOVA with Tukey's HSD multiple comparison post hoc test. Bars represent the mean ± SE. (e, f) Silencing efficiency of g‐C_3_N_4_@dsGFP on endogenous gene expression in 16C. Leaves were sprayed with naked dsGFP, CDs@dsGFP, nanosheet@dsGFP or nanoporous@dsGFP. Samples were collected at 1 and 3 dps after washing with RNase‐free water. (e) Results of RT‐qPCR and (f) results of western blot using GFP antibody. Data represent mean ± SE of ≥3 biological replicates. Different letters indicate significant differences (*P* < 0.05, one‐way ANOVA with Tukey's HSD test). (g) Laser confocal photographs showing the GFP expression by infiltrating naked GFP expression plasmid of pCass4‐Rz‐GFP, g‐C_3_N_4_ CDs@GFP, g‐C_3_N_4_ nanosheet@GFP and g‐C_3_N_4_ nanoporous@GFP. From left to right, treatments included naked GFP expression plasmid, g‐C_3_N_4_‐CDs@GFP, g‐C_3_N_4_ nanosheet@GFP and g‐C_3_N_4_ nanoporous@GFP. The first row shows confocal microscopy images taken at 1 dps. The second row shows images taken at 3 dps. The scale bar represents 50 μm. (h) Statistical comparison of GFP fluorescence intensity from different treatments, including GFP expression plasmid, g‐C_3_N_4_ CDs@GFP, g‐C_3_N_4_ nanosheet@GFP and g‐C_3_N_4_ nanoporous@GFP. Each treatment consisted of six leaves, each of which was photographed in five randomly selected locations. Different letters above each bar indicate significant differences at *P* < 0.05 as determined by one‐way ANOVA with Tukey's HSD multiple comparison post hoc test. Bars represent the mean ± SE.

Second, the localization of Cy_3_ labelled dsRNA on g‐C_3_N_4_ nanosheet, g‐C_3_N_4_ nanoporous and g‐C_3_N_4_ CDs onto the plant cell was investigated as described in the Materials and Methods. After leaves were washed with water at 24 h post infiltration, CLSM (Figure 2a) showed that the red fluorescence signal of g‐C_3_N_4_ CDs@dsCP‐Cy_3_, nanosheet@dsCP‐Cy_3_ and nanoporous@dsCP‐Cy_3_ overlapped with the green fluorescence of intracellular GFP to some extent, whereas there was no red signal of free dsCP‐Cy_3_. This result supported that dsRNA on g‐C_3_N_4_ nanomaterials could enter plant cells after spraying. The red fluorescence signal of g‐C_3_N_4_ CDs@dsCP‐Cy_3_ was clearly observed along the cell contour with more overlapping signal areas than the two other g‐C_3_N_4_ nanomaterials (Figure 2a), implying that g‐C_3_N_4_ CDs have greater potential for nucleic acid delivery capability.

To further investigate the internalization of g‐C_3_N_4_@dsCP by plant cells and its subcellular distribution, we also collected treated *N. benthamiana* leaf tissue at 24‐h post infiltration, fixed it, sectioned it and imaged it by TEM. It was found that structures such as nanomaterials were absent in non‐infiltrated areas and dsCP‐infiltrated areas, whereas these structures were common in g‐C_3_N_4_@dsCP‐infiltrated areas between the cell wall and plasma membrane (Figure 2b). These results suggest that g‐C_3_N_4_ CDs have greater potential and efficiency in penetrating the plant cell wall than the other two g‐C_3_N_4_ nanomaterials.

Finally, the capability and efficiency of delivering exogenous nucleic acids into plant cells were subsequently verified by GFP silencing via 16C and GFP expression in *N. benthamiana* through the delivery of dsRNA and a GFP expression plasmid (pCass4‐Rz‐GFP), respectively. For the GFP silencing experiment, free dsGFP, CDs@dsGFP, nanosheet@dsGFP and nanoporous@dsGFP at a nanomaterial:dsGFP ratio of 4:1 were sprayed on *N. benthamiana* 16C. The GFP expression levels of the treated leaves in 16C were detected by CLSM (Figure 2c,d), RT‐qPCR (Figure 2e) and western blot (Figure 2f) at 1 and 3 dps. The CLSM, RT‐qPCR and western blot results (Figure 2d–f) revealed that spraying CDs@dsGFP, nanosheet@dsGFP and nanoporous@GFP significantly inhibited the fluorescence intensity of 16C GFP to different degrees compared with that of the control. One day after spraying, the GFP transcriptional level of the CDs@dsGFP, nanosheet@dsGFP and nanoporous@GFP groups was reduced by 40.56%, 46.02% and 17.46%, respectively, compared to the dsGFP‐treatment group (Figure S4). Three days after spraying, the GFP transcriptional level of the CDs@dsGFP, nanosheet@dsGFP and nanosheet@GFP treatments was reduced by 45.26%, 31.84% and 2.90%, respectively, compared to the dsGFP‐treatment group (Figure S4). On the other hand, pCass4‐Rz‐GFP was loaded on CDs, g‐C_3_N_4_ nanosheet and nanoporous through electrostatic adsorption, as with dsRNA. These three different g‐C_3_N_4_@GFP materials were subsequently infiltrated into the plant leaves of *N. benthamiana* via injection. CLSM revealed that CDs@GFP infiltration obviously resulted in significant overexpression of the GFP gene at 1 and 3 dps. The ability of nanosheet@GFP and nanoporous@GFP to express GFP was weaker than that of CDs@GFP at 1 and 3 dps (Figure 2g,h). These results showed that the CDs morphology of g‐C_3_N_4_ has better nucleic acid delivery ability than the sheet and nanoporous morphologies; this may be due to the stronger ability of CDs to penetrate the cell wall, which is supported by the localization detection of the g‐C_3_N_4_ (Figure S4) and g‐C_3_N_4_@dsRNA (Figure 2a,b).

### Delivery efficiency of exogenous nucleic acid by g‐C_3_N_4_ @dsRNA is regulated by stomatal and endocytosis pathways in plants

**Figure 3:**
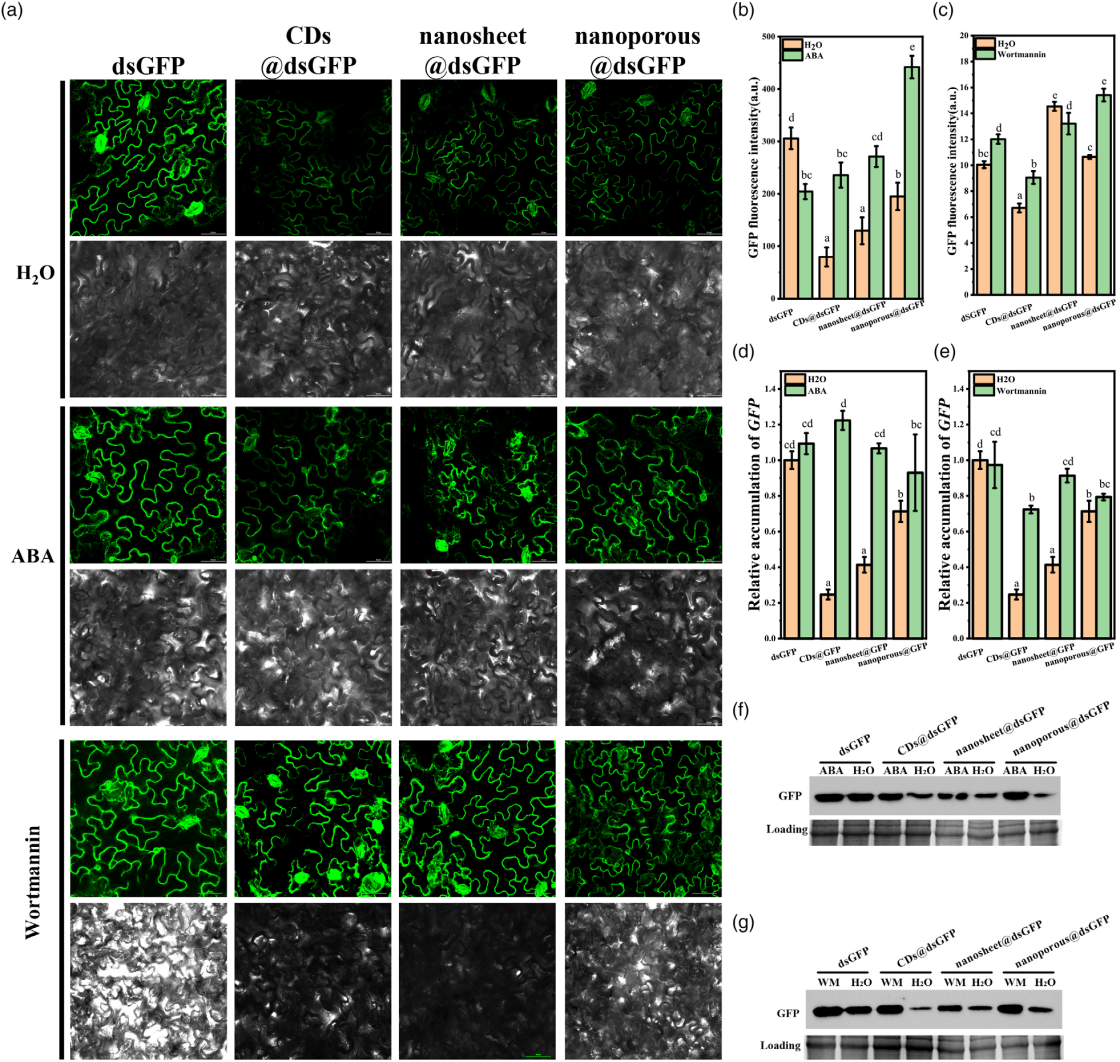
The delivery ability of g‐C_3_N_4_ with different morphologies under ABA‐ or wortmannin‐treatment on plants. (a) Leaves were treated with water, ABA (via spraying), or wortmannin (via headless syringe infiltration) for 4 h. Laser confocal microscopy images were taken to observe GFP fluorescence in 16C leaves 24 h after treatment with dsGFP, CDs@dsGFP, nanosheet@dsGFP and nanoporous@dsGFP. The scale bar represents 50 μm. (b, d and f) Impact of ABA on g‐C_3_N_4_@dsGFP‐mediated interference in endogenous gene expression. (b) Variations in GFP fluorescence intensity, (d) transcript levels of GFP and (f) protein abundance. Assessment of ABA's impact on RNA interference efficiency following treatment with either water or ABA and subsequent application of: dsGFP, CDs@dsGFP, nanosheet@dsGFP or nanoporous@dsGFP. Each treatment consisted of six leaves, each of which was photographed in five randomly selected locations. The fluorescence signal intensity of the images was quantified and analysed using ImageJ software. Different letters above each bar indicate significant differences at *P* < 0.05 as determined by one‐way ANOVA with Tukey's HSD multiple comparison post hoc test. Bars represent the mean ± SE. (c, e and g) Impact of wortmannin on g‐C_3_N_4_@dsGFP‐mediated interference in endogenous gene expression. (c) Variations in GFP fluorescence intensity, (e) transcript levels of GFP and (g) protein abundance. Assessment of wortmannin's impact on RNA interference efficiency following treatment with either water or wortmannin and subsequent application of: dsGFP, CDs@dsGFP, nanosheet@dsGFP or nanoporous@dsGFP. Each treatment consisted of six leaves, each of which was photographed in five randomly selected locations. The fluorescence signal intensity of the images was quantified and analysed using ImageJ software. Different letters above each bar indicate significant differences at *P* < 0.05 as determined by one‐way ANOVA with Tukey's HSD multiple comparison post hoc test. Bars represent the mean ± SE.

In addition to the nanomaterial, the impact of the plant state on the efficiency of g‐C_3_N_4_@dsRNA delivering exogenous nucleic acids is an important issue. First, the effects of stomatal opening and closing on g‐C_3_N_4_@dsRNA delivery of exogenous nucleic acids during g‐C_3_N_4_@dsRNA spraying were investigated by evaluating the silencing effect of g‐C_3_N_4_@dsGFP on GFP gene expression at 16C. As a plant hormone that plays a crucial role in enhancing drought resistance, ABA can facilitate stomatal closure in plants (Hsu *et al*., 2021). By contrast, abundant water in the plant root soil could induce stomatal opening in 16C (Figure S5a). At the same time, ABA treatment of 16C for 4 h could significantly decrease the stomatal aperture (Figure S5b). The g‐C_3_N_4_@dsGFP‐induced GFP silencing in the ABA treatment group was significantly weaker than that in the water treatment group (Figure 3a,b,e,g). All three different morphologies of g‐C_3_N_4_@dsGFP were this case. These results showed that the opening and closing of plant stomata affected the efficiency of exogenous nucleic acid delivery after spraying g‐C_3_N_4_@dsRNA. Stomatal opening may facilitate the entry of nanomaterials into the inner tissue of plant leaves.

On the other hand, endocytosis provides a major route of entry for membrane proteins, lipids and extracellular molecules into plant cells (Fan *et al*., 2015). Wortmannin is a widely used pharmaceutical compound that is employed to define vesicular trafficking routes of particular proteins or cellular compounds (Takáč *et al*., 2012). After 16C leaves were treated with wortmannin for 4 h, g‐C_3_N_4_@dsGFP was sprayed to detect the efficiency of GFP silencing in 16C leaves, thereby eliminating the effects of pores. Compared with the results of the control group, which was injected with water, the injection of wortmannin greatly reduced the efficiency of delivering exogenous dsGFP by all g‐C_3_N_4_@dsGFP into plant cells to silence GFP (Figure 3a,c,f,h). These results showed that the delivery efficiency of exogenous dsRNA by the g‐C_3_N_4_ nanosystem was affected by the endocytosis pathway of the plant cells.

### Application of g‐C_3_N_4_ @dsRNA spray for protection against plant virus‐related diseases

Given the absence of effective antiviral drugs, RNA interference (RNAi) represents a potent strategy for combating plant virus infections. However, the conventional approach of delivering siRNA into intact plants via Agrobacterium or viruses is species‐restricted, intricate and time‐consuming, thereby restricting the application of RNAi against viral diseases. Our previous results revealed that g‐C_3_N_4_@dsRNA is a novel and efficient nucleic acid delivery material. Thus, we further tested whether g‐C_3_N_4_@dsRNA could be used for the prevention and control of plant viral diseases. The model virus–plant interaction system, TMV*–N. benthamiana*, was chosen in our study because it is an ideal test object.

First, dsRNA needs to be prepared in large quantities and at low cost in plant disease protection applications. Thus, dsRNA, which can specifically target the key genes *CP*, *MP*, *RdRp* and *PR* from the TMV virus, was synthesized by transforming the expression vector into *E. coli* HT115 (RNase mutant strain) and inducing it with IPTG, according to a previous study, as shown in Figure S6a (Ganbaatar *et al*., 2017). Our RT‐qPCR (Figure S6b) and agarose gel electrophoresis (Figure S6c) results revealed that the optimum working concentration of IPTG was 4 mM in dsRNA generation. Although dsCP, dsMP, dsRdRp and dsPR loaded onto nanomaterials are total RNA expressed by bacteria, not pure dsRNA, the preparation of dsRNA using this method is very inexpensive and is more consistent with the requirements of production applications. All these different types of crude dsRNA from bacteria were then loaded onto nanosheets, nanopores and CDs of g‐C_3_N_4_, and the loading ratios of all g‐C_3_N_4_ to dsRNAs were maintained at 4:1 (W: W) to make sure all the RNA is completely loaded onto the nanomaterial (Figure 1e–g).

**Figure 4:**
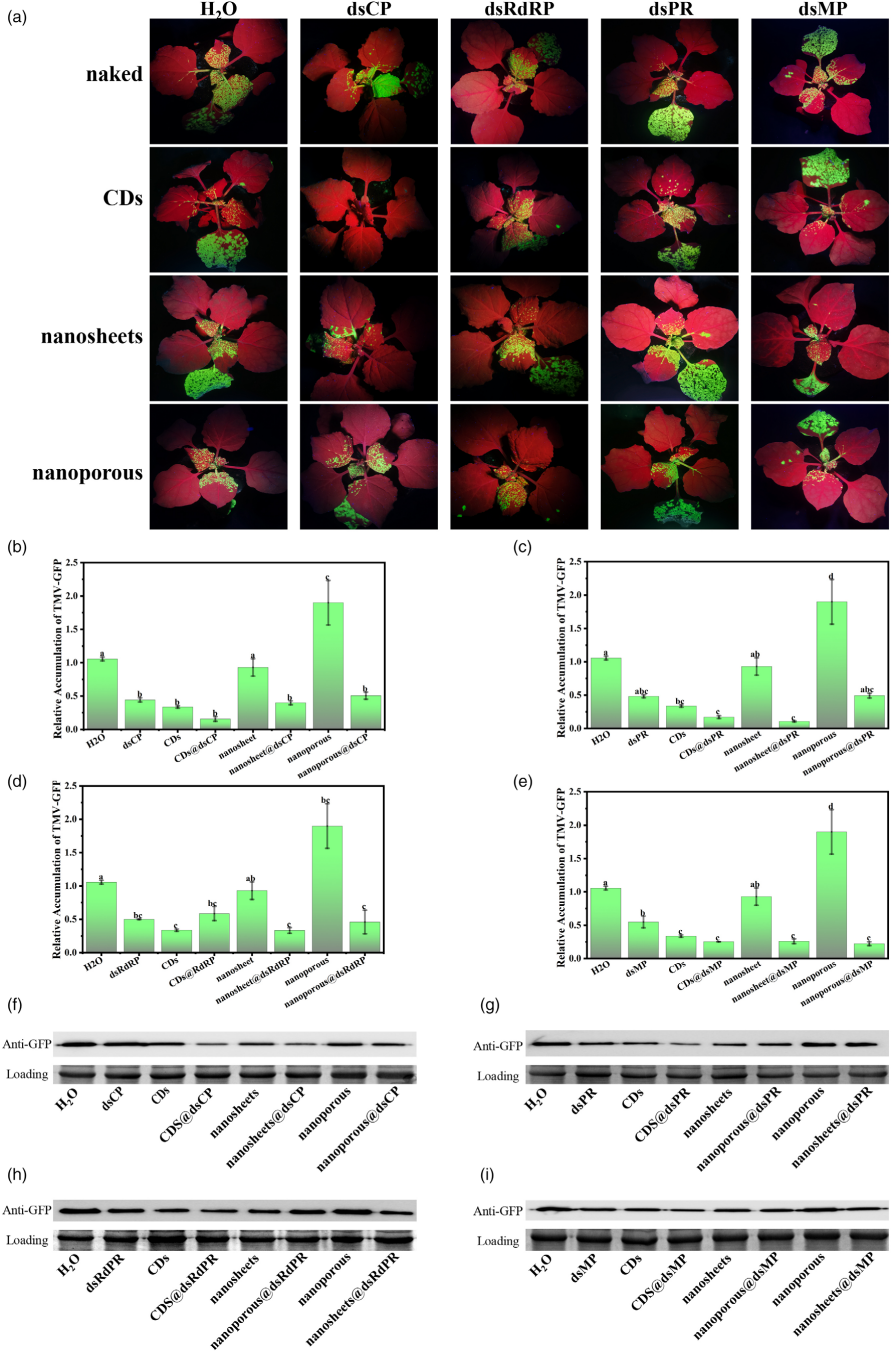
g‐C_3_N_4_ delivery of exogenous dsRNA to achieve TMV virus disease prevention. (a) Phenotypes of TMV‐GFP infection in *N. benthamiana*. Leaves were treated with naked dsCP, naked dsPR, naked dsRdPR, naked dsMP, g‐C_3_N_4_ CDs, g‐C_3_N_4_ CDs@dsCP, g‐C_3_N_4_ CDs@dsPR, g‐C_3_N_4_ CDs@dsRdPR, g‐C_3_N_4_ CDs@dsMP, g‐C_3_N_4_ nanosheet, g‐C_3_N_4_ nanosheet@dsCP, g‐C_3_N_4_ nanosheet@dsPR, g‐C_3_N_4_ nanosheet@dsRdPR, g‐C_3_N_4_ nanosheet@dsMP, g‐C_3_N_4_ nanoporous, g‐C_3_N_4_ nanoporous@dsCP, g‐C_3_N_4_ nanoporous@dsPR, g‐C_3_N_4_ nanoporous@dsRdPR and g‐C_3_N_4_ nanoporous@dsMP, followed by inoculation with TMV‐GFP at 1 dps. Phenotypic observations were recorded and samples were collected at 7‐day post inoculation (dpi). (b–e) The accumulation of TMV‐GFP for each treatment was quantified by RT‐qPCR. Different letters above each bar indicate significant differences at *P* < 0.05 as determined by one‐way ANOVA with Tukey's HSD multiple comparison post hoc test. Bars represent the mean ± SE. (f–i) Western blot detection of the GFP protein content of TMV‐GFP for different treated plants at 7 dpi. Loading control for Coomassie bright blue staining. All experiments were repeated at least three times with similar results.

TMV‐GFP virus was inoculated on *N. benthamiana* after spraying different g‐C_3_N_4_@dsRNAs for 1 day. Water and different naked crude dsRNAs were used as controls. After 7 days of TMV‐GFP inoculation, the results of ultraviolet lamp photography (Figure 4a) revealed that the g‐C_3_N_4_ CDs@dsCP treatment nearly completely suppressed the TMV‐GFP virus, showing the best virus‐inhibiting effect among all the different examined treatments. TMV‐GFP accumulation detected by RT–qPCR (Figure 4b–e) and immunoblotting (Figure 4f–i) revealed that the accumulation of TMV‐GFP decreased to different degrees after different g‐C_3_N_4_@dsRNA treatments. For g‐C_3_N_4_ with different morphologies loaded with the same amount of dsRNA, the ability of g‐C_3_N_4_@dsRNA to inhibit TMV‐GFP virus was in the order of CDs > sheets > nanopores (Figure 4), which also corresponded with the ability of g‐C_3_N_4_@dsRNA to deliver exogenous nucleic acids (Figure 2c–f). For dsRNAs targeting different TMV genes loaded with g‐C_3_N_4_ with the same morphology, the ability of g‐C_3_N_4_@dsCP to inhibit TMV‐GFP virus was greater than that of g‐C_3_N_4_@dsRdRP, g‐C_3_N_4_@dsMP and g‐C_3_N_4_@dsPR, which was consistent with the results of naked RNA treatment (Figure 4). We also found that g‐C_3_N_4_ CDs and g‐C_3_N_4_ sheets could also inhibit the accumulation of TMV‐GFP compared with water treatment.

Following the evaluation of antiviral effects using different target genes and material combinations, we identified that CP‐targeted RNAi exhibited the highest antiviral efficiency. To investigate whether the three g‐C_3_N_4_ could effectively deliver dsCP into plant cells and subsequently generate siRNA for antiviral defence, we conducted spray application experiments on *N. benthamiana* leaves. Total RNA extracted at 1 and 3 dps was analysed by northern blotting (Figure S7). The results demonstrated that all three g‐C_3_N_4_ successfully delivered dsCP into plant cells and mediated siRNA production (Figure S7). Notably, at 3 dps, the CDs@dsCP complex generated significantly higher levels of siRNA compared to the other two materials.

To further examine whether g‐C_3_N_4_ CDs@dsCP can provide prolonged protection to *N. benthamiana*, single treatments of water, dsCP, CDs or CDs@dsCP were applied with TMV‐GFP at 1, 3 or 5 dps. After 7 days of TMV‐GFP inoculation, ultraviolet lamp photography (Figure S8a), RT‐qPCR (Figure S8b) and western blot (Figure S8c) were used to detect the presence of TMV‐GFP in the apical leaves. Our results showed that g‐C_3_N_4_ CDs and g‐C_3_N_4_ CDs@dsCP could all inhibit TMV‐GFP virus in the apical leaves after a single spray for 1 day. Comparisons between different treatments revealed that g‐C_3_N_4_ CD@dsCP treatment inhibited TMV‐GFP virus in the apical leaves even after a single spray of 5 days, whereas the dsCP and g‐C_3_N_4_ CDs treatments retarded the virus only after the first day of treatment. We also observed this phenomenon in *C. annuum L*. As shown in Figure S9, the g‐C_3_N_4_ CD@dsCP treatment could also significantly inhibit TMV‐GFP virus after a single spray of 5 days on *C. annuum L*. These results showed that g‐C_3_N_4_ CDs@dsCP can provide protection in a systemic host plant against TMV‐GFP for at least 5 days.

### Impacts of g‐C_3_N_4_ @dsRNA for plant growth

**Figure 5:**
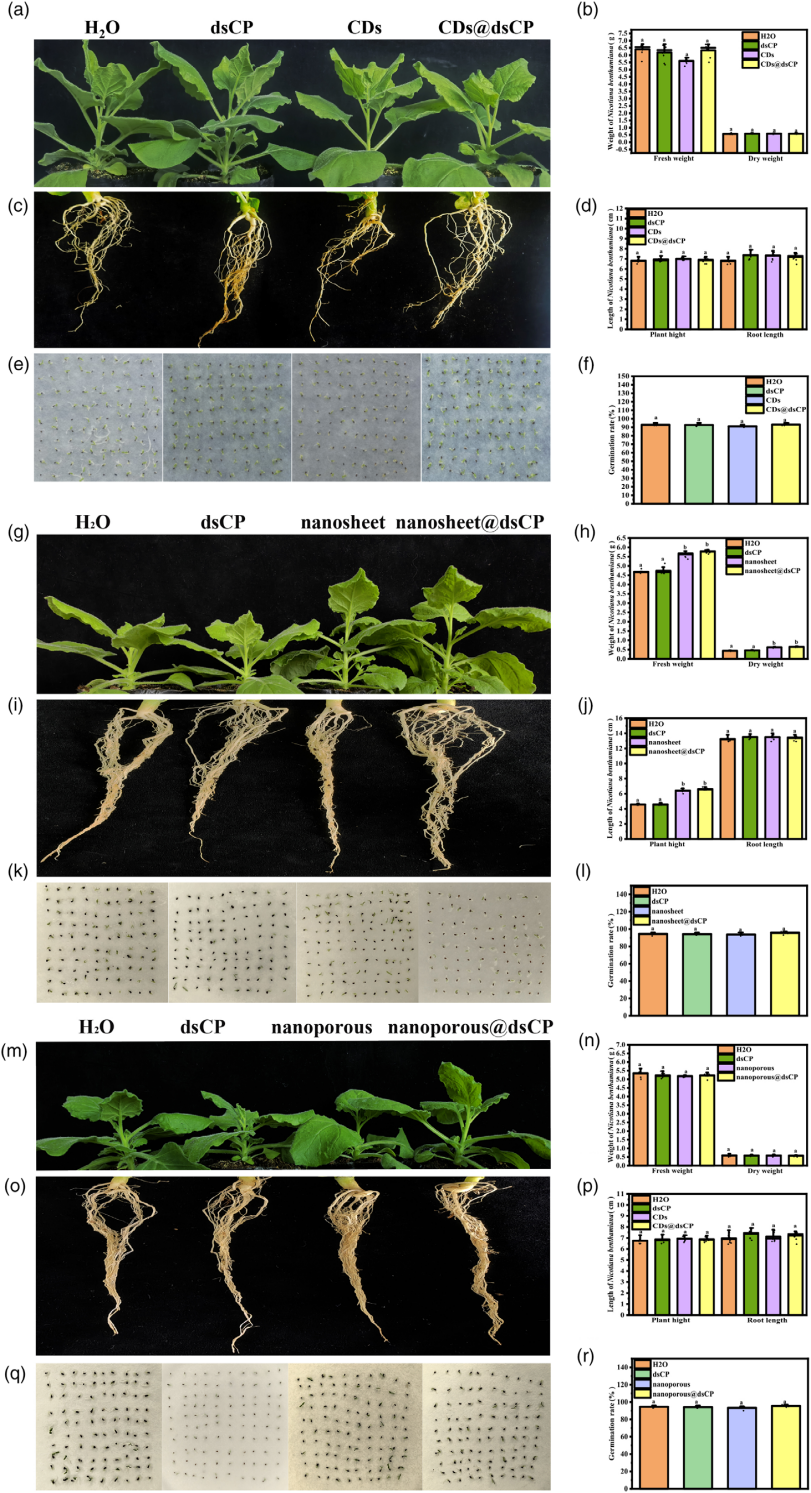
Evaluation of effects of dsCP, g‐C_3_N_4_ and g‐C_3_N_4_@dsCP on plant growth. (a**–**f) Evaluation of effects of dsCP, CDs and CDs@dsCP on plant height, root length, fresh weight, dry weight and germination rate. (a) Effects of nanomaterials on plant height. Leaves of 2‐week‐old *N. benthamiana* plants were sprayed with H_2_O, dsCP, CDs or CDs@dsCP. Photographs of plant height were taken on the 15th day after treatment. Each treatment group included at least three biological replicates. (b) Statistical analysis of fresh weight and dry weight of *N. benthamiana* under different treatments. Different letters above each bar indicate significant differences at *P* < 0.05 as determined by one‐way ANOVA with Tukey's HSD multiple comparison post hoc test. Bars represent the mean ± SE. (c) Effects of nanomaterials on root length. Leaves of 2‐week‐old *N. benthamiana* plants were sprayed with H_2_O, dsCP, CDs or CDs@dsCP. Photographs of root length were taken on the 15th day after treatment. Each treatment group included at least three biological replicates. (d) Statistical analysis of plant height and root length of *N. benthamiana* under different treatments. Different letters above each bar indicate significant differences at *P* < 0.05 as determined by one‐way ANOVA with Tukey's HSD multiple comparison post hoc test. Bars represent the mean ± SE. (e) Germination rate of *N. benthamiana* under different treatments. Seeds of *N. benthamiana* were soaked in H_2_O, dsCP, CDs or CDs@dsCP for 12 h. After soaking, the seeds were placed on moist filter paper for further treatment. Photographs were taken, and germination rates were recorded on the fourth day. (f) Statistical analysis of germination rate of *N. benthamiana* under different treatments. Different letters above each bar indicate significant differences at *P* < 0.05 as determined by one‐way ANOVA with Tukey's HSD multiple comparison post hoc test. Bars represent the mean ± SE. (g–l) Evaluation of effects of dsCP, nanosheet and nanosheet@dsCP on plant height, root length, fresh weight, dry weight and germination rate. Materials were processed and data were collated using a similar methodology as the H_2_O, CDs and CDs@dsCP experiments. (m–r) Evaluation of effects of the H_2_O, dsCP, nanoporous and nanoporous@dsCP on plant height, root length, fresh weight, dry weight and germination rate. Materials were processed and data were collated using the similar methodology as H_2_O, CDs and CDs@dsCP experiments.

For the protective effect of different g‐C_3_N_4_ and g‐C_3_N_4_@dsRNAs on plant viral disease, there were no symptoms of toxicity after spraying dsRNA, g‐C_3_N_4_ or g‐C_3_N_4_@dsRNA. No differences in the fresh weight, dry weight, plant height or root length of *N. benthamiana* were observed after being sprayed with water, dsCP, CDs, CDs@dsCP, nanoporous or nanoporous@dsCP (Figure 5a–d,m–p). However, the fresh and dry weights and plant height of nanosheet and nanosheet@dsCP were significantly higher than those of the control, but there was no significant difference in root length (Figure 5g–j). Seed germination is widely acknowledged to exhibit heightened sensitivity to toxic substances (Begum *et al*., 2024; Luo *et al*., 2024). Thus, to further evaluate the potential effects on plants, we also examined the effects of dsCP, g‐C_3_N_4_ and g‐C_3_N_4_@dsCP on the germination rate of *N. benthamiana* seeds. As shown in Figure 5e,f,k,l,q,r, compared with the water treatment, the dsCP, CDs and CDs@dsCP nanosheet, nanosheet@dsCP, nanoporous and nanoporous@dsCP did not significantly affect seed germination. Thus, the spray of g‐C_3_N_4_@dsRNA developed in our work offers a safe approach for plant exogenous nucleic acid delivery and viral protection, circumventing the limitations associated with conventional transgenic methods and enabling precise targeted treatment of plant viral diseases in the absence of effective control agents.

In addition, the biosafety evaluation of g‐C_3_N_4_@dsCP and related nanocomposites using zebrafish (aquatic model) and earthworms (soil model) demonstrated no observed mortality after 96 h of exposure to 20 μg·mL^−1^ of dsCP, CDs, CDs@dsCP, nanosheet, nanosheet@dsCP, nanoporous or nanoporous@dsCP in zebrafish tests (100% survival), with earthworms similarly showing 100% survival after 48 h of exposure at the same concentration (Figure S10). According to toxicity criteria (LC_50_ > 10 μg·mL^−1^ is low toxicity), all tested materials exhibited low acute toxicity to both aquatic and soil organisms, confirming the environmentally benign characteristics of these g‐C_3_N_4_@dsCP complexes (Dai *et al*., 2024).

## Discussion

### Features and advantages of our novel carbon‐based nucleic acid delivery system

**Figure 6:**
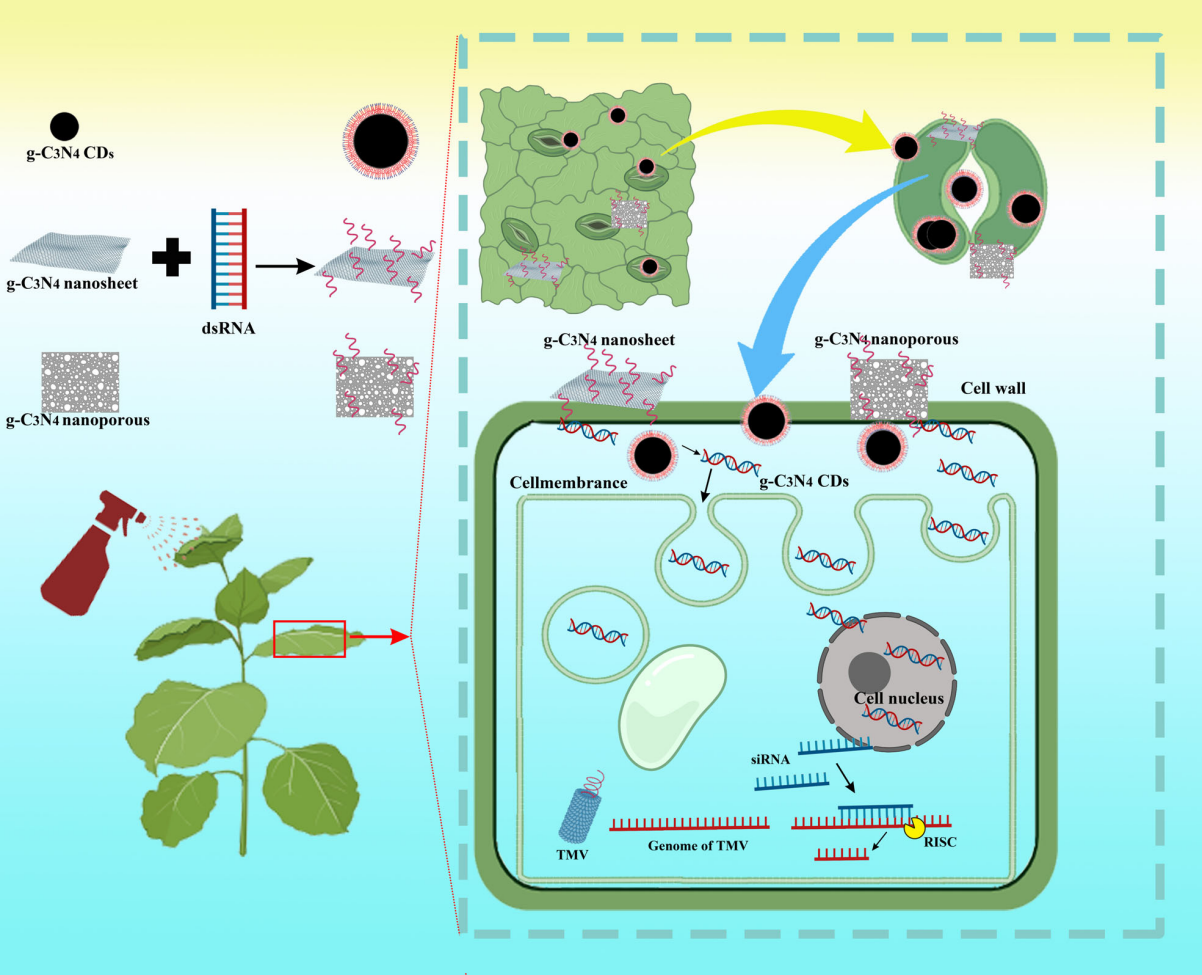
Frame diagram of this study.

In our previous studies, g‐C_3_N_4_ was recognized for its antibacterial and anoomycete properties when applied to plants (Cai *et al*., 2021a,b). As a carbon‐based nanomaterial with photocatalytic activity, g‐C_3_N_4_ has been demonstrated to have beneficial effects in photocatalysis, electrocatalysis and heterogeneous catalysis due to its ease of synthesis, low cost, low toxicity and abundance of electrons (Zhu *et al*., 2014). Here, we demonstrated the feasibility of the use of three different morphologies of g‐C_3_N_4_ nanomaterials (Figure 1) with nucleic acids for the transport of exogenous nucleic acids into intact plant cells (Figure 2) and achieve excellent antiviral function (Figure 4) when g‐C_3_N_4_ nanomaterials are loaded with dsRNA targeting viral genes. For these three different morphologies of g‐C_3_N_4_ nanomaterials, g‐C_3_N_4_ CDs can even be sprayed to achieve dsRNA and plasmid delivery into mature plant cells. To the best of our knowledge, the nanomaterial for plant RNA delivery remains remarkably limited—while thousands of nanostructures exist, only a few nanomaterials have been validated for intact plant cell delivery (Cai *et al*., 2024; Demirer *et al*., 2020; Hao *et al*., 2024; Li *et al*., 2022; Mitter *et al*., 2017; Wang *et al*., 2023; Zhang *et al*., 2022a). Here, we propose a novel carbon‐based nanomaterial that efficiently delivers exogenous nucleic acids into mature plant cells even by spraying, which can expand the range of options for nucleic acid delivery to mature plants (Figure 6). In our study, g‐C_3_N_4_ nanomaterials were synthesized using urea as a single precursor without producing toxic compounds, as described in previous reports (Cai *et al*., 2021b; Zhang *et al*., 2013a; Zhou *et al*., 2013). Our g‐C_3_N_4_ nanosystem offers significant advantages in terms of plant safety and production costs. The CDs, CDs@dsRNA, nanoporous and nanoporous@dsCP showed no effects on plant growth and seed germination. Our results demonstrate that nanosheet and nanosheet@dsCP positively regulate plant growth, significantly promoting stem elongation and enhancing dry biomass accumulation (Figure 5), which aligns with previous findings by Cai *et al*. (2021a,b).

The biosafety of nanomaterials is of paramount importance, particularly for agricultural applications where low toxicity and environmentally friendly materials are essential. To evaluate our nanomaterials' safety profile, we conducted comprehensive biosafety assessments using two ecologically relevant model organisms: zebrafish (aquatic model) and earthworms (soil model). Our results demonstrate that both the synthesized g‐C_3_N_4_ and g‐C_3_N_4_@dsCP showed no acute toxicity at concentrations up to 20 μg/mL in either organism (Figure 6). These findings confirm the environmental compatibility of g‐C_3_N_4_, aligning with previous safety reports (Shen *et al*., 2025; Wang *et al*., 2024). Collectively, these results provide critical safety data to support the potential agricultural applications of these nanomaterials (Liu *et al*., 2025).

In our current work, we synthesized g‐C_3_N_4_ with different morphologies and structures using urea as the precursor. Considering that CDs can deliver dsRNA by spraying, we believe that g‐C_3_N_4_ CDs@dsRNA is a set of RNA pesticides with great potential in the control of plant viral diseases. The difference in the dsRNA binding ability of the nanomaterials was analysed via gel‐based analysis, revealing that the g‐C_3_N_4_ CDs had the greatest ability to load nucleic acids, while nanoporous g‐C_3_N_4_ showed the least ability. The properties of nanomaterials are critical for the delivery of exogenous nucleic acids into plants for crop protection (Qiao *et al*., 2023; Zhang *et al*., 2022a). Delgado‐Martín and Schwartz *et al*. demonstrated that carbon dots synthesized from sucrose and glucose precursors through a one‐step PEI‐assisted hydrothermal method exhibited superior dsRNA delivery efficiency and successfully silenced endogenous genes in both *N. benthamiana* and tomato (Delgado‐Martín *et al*., 2022; Schwartz *et al*., 2020). Notably, the positive charge of these carbon dots enhanced foliar adsorption and facilitated systemic movement of RNAi signals throughout the plant. Our g‐C_3_N_4_ nanomaterial also showed good ability to protect dsRNA from degradation by RNA enzymes, which was similar to many previous nanosystems (Demirer *et al*., 2020; Hao *et al*., 2024; Li *et al*., 2022; Mitter *et al*., 2017; Wang *et al*., 2023; Zhang *et al*., 2022a).

In this study, we also found that all three g‐C_3_N_4_ species could stick to the plant cell surface (Figure 2). It may be due to their overall positive charge, which led them to be easily adsorbed on the negatively charged cell wall. Moreover, the g‐C_3_N_4_ CDs easily broke through the cell wall and entered the plant cells (Figure 2). Due to the limitations of the current observation techniques, our results are supported only by CLSM and TEM.

### Effects of g‐C_3_N_4_ ‐mediated nucleic acid delivery into plant cells

The inherent impermeability of plant cell walls presents a fundamental challenge for exogenous nucleic acid delivery, as they effectively block macromolecular transport through their rigid polysaccharide matrix (Hou *et al*., 2021; Wang *et al*., 2016b, 2019; Zhang *et al*., 2021). To date, only a limited number of engineered nanomaterials have succeeded in overcoming these barriers. These nanomaterials achieve cellular entry through precisely controlled physicochemical optimization—including size modulation, surface charge engineering and functional group modification (Li *et al*., 2016; Ma *et al*., 2016). For instance, carbon nanotubes (CNTs) utilize their high aspect ratio and specific surface area to bypass plant cell wall barriers: Single‐walled carbon nanotubes (SWNTs) enter plant cells via a temperature‐dependent, clathrin‐mediated endocytosis mechanism, while multi‐walled carbon nanotubes (MWNTs) passively deliver nucleic acids or drugs across cell membranes (Law *et al*., 2022; Serag *et al*., 2013; Vashist *et al*., 2011).

Prior to this study, very little had been known about the influence of the different morphological structures of the delivery materials on their nucleic acid delivery capabilities. Our comparative analysis reveals distinct morphological advantages of g‐C_3_N_4_ CDs over nanoporous and nanosheet architectures in plant biomolecule delivery. Specifically, CDs demonstrated superior dsRNA and plasmid transport efficiency into intact plant cells (Figure 2), consistent with recent findings in gold nanoparticle (Au NP) systems where reduced dimensions enhanced cell wall interaction kinetics and delivery persistence (Zhang *et al*., 2022a). Apart from the morphological advantages of g‐C_3_N_4_ CDs, the enhanced delivery capability of CDs can be attributed to their optimized surface characteristics and nanoscale dimensions. With abundant carboxyl groups generating high surface potential (−32.7 ± 1.5 mV), CDs exhibited exceptional dsRNA loading capacity (Figure 1c,i) while providing effective nucleic acid protection against RNase degradation (Figure 1l). These charged nanostructures demonstrated improved phyllosphere adhesion (Zhou *et al*., 2023), potentially increasing their residence time and delivery opportunities on plant surfaces. Notably, our observations on the distribution of nanomaterials around plant cells reveal size‐dependent transport differences: CDs@dsRNA with a diameter smaller than 20 nm (Figure 1a) can penetrate the cell wall and reach the cell wall–membrane interface, while larger nanosheets@dsRNA and nanoporous@dsRNA composites are still confined to the periplasmic region (Figure 2). These morphological, dimensional and surface characteristics make it possible for CDs to achieve highly efficient gene silencing through foliar spraying.

Apart from the inherent properties of the materials themselves, we hypothesize that the plant state plays a role in the efficiency of nucleic acid delivery by g‐C_3_N_4_. Our results showed that plant stomata can not only regulate the entry of exogenous bacteria into the leaf tissue but also the exogenous nucleic acid delivery nanomaterials (Figure 3), which is consistent with previous speculations (Wu and Li, 2022). Furthermore, the 68% reduction in endogenous gene silencing efficiency following wortmannin pretreatment (Figure 3) strongly suggests endocytic involvement in CD internalization, contrasting with conventional receptor‐mediated pathways in animal models (Wolfram and Ferrari, 2019). All these findings establish morphology‐engineered carbon nanomaterials as versatile platforms for plant genetic manipulation, combining protective cargo encapsulation with multiple internalization routes.

### Characterization of g‐C_3_N_4_ @dsRNA for plant virus control

Since RNAi was discovered in the nematode *Caenorhabditis elegans* (a breakthrough awarded the Nobel Prize in 2006), it has not only revolutionized functional genomics research but also emerged as a powerful tool for crop protection (Gordon and Waterhouse, 2007). In plant pathology specifically, RNAi technology targeting essential genes of pests and pathogens has demonstrated tremendous potential to advance sustainable agriculture (Hou *et al*., 2019; Padilla‐Roji *et al*., 2023; Wang *et al*., 2016a; Yu *et al*., 2016; Zhang *et al*., 2017). Building on this foundation, dsRNA, which is processed into siRNA, has proven effective against multiple economically important viruses (Holeva *et al*., 2021; Konakalla *et al*., 2016, 2021; Tenllado *et al*., 2003; Xu *et al*., 2023).

The practical application of exogenous dsRNA for viral control faces significant challenges due to the ubiquitous presence of RNase A in the environment and the inherent impermeability of plant cell walls. Foliar application of dsRNA induces sequence‐specific RNA interference (RNAi) against Tomato mosaic virus (ToMV) in tomato plants, but this antiviral effect exhibits clear dose dependency, requiring a minimum threshold of 200 μg for effective viral suppression (Rego‐Machado *et al*., 2020). Therefore, the efficacy of RNAi‐based strategies largely depends on efficient RNA delivery systems and the RNase enzyme protection ability of nanomaterials. As demonstrated in our study, g‐C_3_N_4_‐loading dsRNA maintains significantly greater stability against RNase degradation compared to free dsRNA, thereby enhancing its bioavailability and antiviral efficacy (Figure 1g–l). Several recent reports (Hao *et al*., 2024; Xu *et al*., 2023) have shown that nanocarriers can overcome plant cellular barriers, significantly improving exogenous RNA uptake. A prime example is the successful use of layered double hydroxide (LDH) nanoparticles for dsRNA delivery in peanut plants (Jiang *et al*., 2024).

Intriguingly, under standardized conditions (4:1 g‐C_3_N_4_‐to‐dsRNA ratio), we observed distinct antiviral efficacies among different g‐C_3_N_4_, which correlated directly with their exogenous nucleic acid delivery capacities. Intriguingly, when targeting different TMV genomic regions, dsRNA targeting the coat protein (dsCP) genes demonstrated optimal control efficacy. These findings underscore two critical aspects of RNAi‐mediated antiviral strategies: (1) the target gene selection profoundly influences antiviral effectiveness (Jiang *et al*., 2011; Mitter *et al*., 2017), likely due to the varying functional importance of viral genes in virion assembly and infectivity; and (2) the nanocarrier morphology significantly impacts delivery efficiency. Furthermore, we identified an intrinsic antiviral property of g‐C_3_N_4_, which exhibited independent TMV suppression activity (Figure 4). This secondary antiviral mechanism, distinct from RNAi‐mediated protection, suggests potential synergistic benefits for plant virus control. While the precise inhibitory mechanism of g‐C_3_N_4_ against TMV requires further elucidation, our current findings demonstrate that g‐C_3_N_4_@dsRNA complexes combine both nanomaterial‐mediated viral inhibition and efficient RNAi delivery. Collectively, these results position our g‐C_3_N_4_‐based nucleic acid delivery system as a promising platform for plant viral disease management, offering: (i) shape‐dependent delivery optimization, (ii) target‐specific RNAi efficacy and (iii) inherent nanomaterial antiviral activity. Collectively, these findings demonstrate the potential of g‐C_3_N_4_‐based nucleic acid delivery systems for managing plant viral diseases.

In advancing nanomaterial‐mediated dsRNA delivery systems for plant virus control, two other critical aspects also demand focused investigation: application methodologies and target gene selection. For instance, carbon quantum dots (CQD) loaded with dsRNA exhibited exceptional antiviral efficacy when administered via root soaking (Xu *et al*., 2023); chitosan‐lipid particles (CLPs) and alginate‐lipid particles (ALPs) loaded with dsRNA successfully protected maize against viral infection by silencing the host susceptibility gene *ZmFd3* (Hao *et al*., 2024).

## Materials and methods

### Synthesis of three morphologies of g‐C_3_N_4_

Following the procedures described in previous studies, three different morphologies of graphene carbon nitride were synthesized (Cai *et al*., 2021b; Zhang *et al*., 2013a; Zhou *et al*., 2013). g‐C_3_N_4_ nanosheets and g‐C_3_N_4_ nanoporous materials were synthesized via thermal polymerization (Cai *et al*., 2021b; Wu *et al*., 2015). g‐C_3_N_4_ nanosheets were prepared according to the procedure used in our previous work (Cai *et al*., 2021b). Specifically, urea (10 g, Shanghai Aladdin Biochemical Technology Co., Ltd., China) was dissolved in 15 mL distilled water, and the pH was adjusted to 4 with HCl. The mixture was subsequently placed in a drying oven and dried at 60 °C. The powders were transferred into an alumina crucible with a cover and heated to 550 °C for 4 h at a heating rate of 10 °C/min. After cooling to room temperature, a pale‐yellow powder of g‐C_3_N_4_ nanosheets was obtained. For the preparation of g‐C_3_N_4_ nanoporous materials, melamine (3 g, Shanghai Aladdin Biochemical Technology Co., Ltd., China) was dissolved in 1 M acetic acid solution (40 mL, Shanghai Aladdin Biochemical Technology Co., Ltd., China) which was added slowly, and the mixture was stirred for 10 h. Then, the suspension was evaporated to a white powder at 60 °C for 12 h. Finally, the powder was placed into a crucible and reacted at 550 °C for 4 h at a heating rate of 2.5 °C/min. Following the procedure used in previous work, g‐C_3_N_4_ nanosheets and g‐C_3_N_4_ nanoporous materials were oxidized (Zhu *et al*., 2022). First, 10 M HNO_3_ (50 mL, Shanghai Aladdin Biochemical Technology Co., Ltd, China) was added to a round‐bottom flask, and then, g‐C_3_N_4_ nanosheets (1 g) and g‐C_3_N_4_ nanoporous materials (1 g), respectively, were added. The mixture was reacted at 80 °C for 24 h and cooled naturally to room temperature. The products were separated, washed and dried. The obtained samples were designated g‐C_3_N_4_ nanosheets‐COOH and g‐C_3_N_4_ nanoporous‐COOH, respectively. For the synthesis of g‐C_3_N_4_ CDs, a solid mixture of urea and sodium citrate (Shanghai Aladdin Biochemical Technology Co., Ltd., China) with a mass ratio of 8:10 was fully ground with a mortar and thermally treated at 180 °C in a hydrothermal synthesis reactor for 1 h (Zhou *et al*., 2013). The resulting yellow powder was dissolved and washed with alcohol by centrifugation at 12 000 rpm for 5 min, after which the resulting mixture was dialyzed against pure water for 24 h.

### PEI functionalization of three morphologies of g‐C_3_N_4_

Nanomaterials of g‐C_3_N_4_ nanosheet‐COOH (25 mg), g‐C_3_N_4_ nanoporous‐COOH (25 mg) and g‐C_3_N_4_ CDs (25 mg) were added to RNase‐free water to obtain aqueous suspensions by ultrasonication. PEI (10 mg; Shanghai Yien Chemical Technology Co., Ltd.), NHS (30 mg, Shanghai Aladdin Biochemical Technology Co., Ltd.) and EDC (20 mg, Shanghai Aladdin Biochemical Technology Co., Ltd.) were added to the above mixture and incubated overnight with shaking. The mixture was loaded onto a 50 kDa filter (Beyotime Biotechnology Co., Ltd., China) and centrifuged at 4000 × ***g*** for 10 min to remove the unreacted PEI, and this process was repeated several times. Finally, three PEI‐modified nanomaterials were obtained: g‐C_3_N_4_ nanosheet‐PEI (hereafter abbreviated as nanosheet‐PEI), g‐C_3_N_4_ CDs‐PEI (hereafter abbreviated as CDs‐PEI) and g‐C_3_N_4_ nanoporous‐PEI (hereafter abbreviated as nanoporous‐PEI).

### Characterization of materials

The morphologies of nanosheet‐PEI and nanoporous‐PEI were investigated by transmission electron microscopy (TEM) (JEM‐2100, JEOL Ltd., Japan). The morphology of CDs‐PEI was examined using high‐resolution transmission electron microscopy (HRTEM, JEOL F200, JEOL Ltd., Japan) operating at an acceleration voltage of 200 kV. An instrument was used to determine the zeta potential and DLS of the materials (NanoBrook Omni, Brookhaven). XPS (Thermo Scientific K‐Alpha) was used to characterize the material functional groups. The nanomaterials were subjected to degassing treatment at 120 °C for 6 h under vacuum prior to nitrogen physisorption measurements. The BET specific surface area analysis was performed using an automated surface area analyzer (ASAP2460, Micromeritics, United States) at 77 K with nitrogen as the coolant medium.

### Plasmid construction and synthesis of dsRNA

The specific DNA fragments targeting genes encoding *Coat protein* (*CP*), *Movement protein* (*MP*), *RNA‐dependent RNA polymerase* (*RdRP*), *polymerase* (*PR*) from TMV and *GFP* from 16C (a stable transgenic *Nicotiana benthamiana* [*N. benthamiana*] line carrying the *mGFP5* transgene) were amplified with specific primers (Table S1). These DNA fragments were subsequently inserted into the *Sac*I and *Pst*I sites of the binary vector L4440, which has T7 promoters with forward and reverse transcriptional initiation at both ends of the fragment insertions, respectively. The T7 promoter is induced by isopropyl‐β‐d‐thiogalactoside (IPTG; Beijing Coleball Technology Co., Ltd.), and the two‐end T7 promoter design of L4440 can be used to generate dsRNA. Next, these recombinant plasmids were transformed into HT115 (an RNase‐deficient *Escherichia coli* strain) to synthesize dsRNA. The above‐expressed double‐stranded RNA fragments were named dsCP, dsMP, dsRdRP, dsPR and dsGFP according to the names of their targeted genes. Seven different concentrations (0, 1, 2, 3, 4, 6 and 8 mM) of IPTG were used to evaluate the optimal concentration for dsRNA production by RT‐qPCR and agarose gel electrophoresis. The dsRNA for the nucleic acid loading ratio test and ribonuclease protection tests was synthesized using an In Vitro Transcription Kit (MAXIscript™ T7 Transcription Kit, Thermo Fisher Scientific).

### dsRNA loading on different shapes of g‐C_3_N_4_ nanomaterials and stability tests

A gel retardation assay was first performed to determine the optimal mass ratio for the combination of dsRNA with nanosheet‐PEI, CDs‐PEI and nanoporous‐PEI. The dsRNA was mixed with nanosheet‐PEI, CDs‐PEI and nanoporous‐PEI at various mass ratios (the mass ratios of g‐C_3_N_4_ nanomaterials/dsRNA were 1:10, 1:2, 1:1, 2:1, 3:1, 4:1, 5:1, 6:1, 7:1, 8:1, 9:1 and 10:1, respectively), and the mixture was incubated at room temperature for 10 min by vortex shaking, followed by agarose gel electrophoresis. The agarose gel was imaged and its fluorescence intensity was quantified using Image Lab software (Bio‐Rad).

To confirm the protective effect of g‐C_3_N_4_ on dsRNA, dsGFP (1 μg) was incubated with nanosheet‐PEI, CDs‐PEI and nanoporous‐PEI. RNase A (Tsingke Biotechnology Co., Ltd., China) was added to nanosheet@dsGFP, CDs@dsGFP and nanoporous@dsGFP for 30 min and 60 min, and then detected by agarose gel electrophoresis. In addition, dsGFP was detected by RT‐qPCR at 1, 3 and 5 days after spraying CDs@dsGFP on *N. benthamiana* leaves to determine the stability of dsRNA when loaded onto CDs.

### Plant cultivation

Transgenic *N. benthamiana* 16C, wild‐type *N. benthamiana* and cultivated pepper Tianyu 8 (*Capsicum annuum* L.) were grown in nutritive soil in a greenhouse. The greenhouse maintained the temperature at 22 °C–26 °C (16 h/8 h day/night). All the experiments in this study were performed on the leaves of 4–6‐week‐old plants.

### Subcellular localization assay of g‐C_3_N_4_ and g‐C_3_N_4_ @dsRNA

For subcellular localization of nanosheet, nanoporous and CDs, Cy3‐labelled nanomaterials were prepared by cross‐linking reactions between the amino groups of Cy3 and the respective g‐C_3_N_4_ nanomaterials. These three different g‐C_3_N_4_ materials were, respectively, sprayed using a spray onto leaves of transgenic *N. benthamiana* 16C, which expresses GFP. After incubation for 24 h and washing the leaves with water, the GFP and Cy3 signals were observed by confocal laser scanning microscopy (CLSM) (Nikon A1R Confocal Microscope System, Nikon Instruments Inc., Tokyo, Japan) to detect the subcellular localization of g‐C_3_N_4_.

For subcellular localization of g‐C_3_N_4_@dsCP, dsCP, labelled with Cy3, was loaded onto nanosheet, nanoporous and CDs. These nanomaterials after loading and free dsCP‐Cy_3_ were sprayed on leaves of 16C. After incubation for 24 h and washing the leaves with water, the GFP and Cy3 signals were observed by CLSM to detect the subcellular localization of g‐C_3_N_4_@dsCP‐Cy3.

Moreover, to further understand the distribution of nanomaterials in plant cells, TEM (JEM‐2100, JEOL Ltd., Japan) was used. Three different shapes of g‐C_3_N_4_, namely, nanosheet@dsCP, nanoporous@dsCP and CDs@dsCP, were infiltrated into *N. benthamiana* leaves using a headless syringe for 24 h. After washing the leaves with water, the leaves were subsequently shredded and fixed with 2.5% glutaraldehyde immediately. Next, gradient dehydration was performed with ethanol and acetone dehydration, and the sample was coated with epoxy resin. The sections were subsequently sectioned with an ultramicrotome and stained with lead citrate and uranyl acetate. Finally, TEM was used to detect the g‐C_3_N_4_ nanoparticles.

### Transient silencing and expression by g‐C_3_N_4_ @dsRNA and g‐C_3_N_4_ @plasmid in *N. benthamiana*

To determine the ability of g‐C_3_N_4_@dsRNA to silence endogenous genes, 4‐week‐old 16C seedlings were sprayed with nanosheet@dsGFP (load ratio of 4:1), CDs@dsGFP (load ratio of 4:1) and nanoporous@dsGFP (load ratio of 4:1). Moreover, bare dsGFP was sprayed as a control. At 1 day post spray (dps) and 3 dps, the intensity of the green fluorescence signal emitted by GFP in the treated leaves was observed under CLSM, and the efficiency of GFP silencing was analysed using RT‐qPCR and WB to evaluate the efficiency of g‐C_3_N_4_@dsGFP‐mediated silencing of GFP.

To determine the ability of the nanomaterials to deliver plasmid DNA into plant cells. First, GFP was cloned and inserted into the pCass4‐Rz vector under the control of a double 35S promoter, which was named pCass4‐GFP. This recombinant vector was used to load the g‐C_3_N_4_ nanomaterials by electrostatic adsorption, similar to previous dsRNA methods. Different shapes of g‐C_3_N_4_ nanomaterials and plasmids (pCass4‐GFP) were loaded at a ratio of 4:1 to obtain nanosheet@GFP, CDs@GFP and nanoporous@GFP. These complexes were subsequently injected into the leaves of *N. benthamiana* via syringes, after which the needles were removed. Finally, the expression level of GFP was observed via CLSM after 1 dpi and 3 dpi.

### Evaluation of RNA interference efficiency of ABA and wortmannin

The effects of the stomatal inhibitor ABA and the endocytic inhibitor wortmannin on the uptake of g‐C_3_N_4_@dsGFP and interference with GFP expression levels were investigated. Leaves of 4‐week‐old 16C seedlings were injected with wortmannin (35 μM) using a headless syringe 4 h in advance. Next, naked dsGFP, nanosheet@dsGFP, CDs@dsGFP and nanoporous@dsGFP were sprayed separately on different regions of the same leaf. The uptake experiment was carried out three times. After 1 dpi and 3 dpi, we compared the GFP signal intensity after different treatments using CLSM, and RT‐qPCR and WB were also used to quantify GFP.

Moreover, the inhibition of plant stomata by ABA was employed to investigate the impact of stomatal behaviour on RNA uptake and RNAi efficiency. Specifically, stomatal closure was induced by applying an ABA spray (100 μM) onto 16C leaves 4 h in advance. Then, dsGFP, nanosheet@dsGFP, CDs@dsGFP and nanoporous@dsGFP were sprayed onto the leaves. After 24 h, CLSM was used to detect signals from GFP, enabling analysis of the efficacy of RNA uptake in the plants following stomatal closure. Additionally, a similar procedure was employed to assess the impact of wortmannin and stomatal closure on the gene interference efficiency of dsGFP, nanosheet@dsGFP, CDs@dsGFP and nanoporous@dsGFP. The fluorescence signal intensity of GFP was measured using CLSM after 1 and 3 days, and RT‐qPCR and WB were also used to quantify GFP.

### Nanomaterial pretreatment and virus vaccination

To evaluate the antiviral effects of different materials, *N. benthamiana* was pretreated by spraying with the same amount of nanosheet@dsRNA (load ratio of 4:1), CDs@dsRNA (load ratio of 4:1) and nanoporous@dsRNA (load ratio of 4:1) 1 day in advance. Then, TMV‐GFP was inoculated by mechanical friction. Moreover, distilled water was used as a control. After inoculation with TMV‐GFP, virus symptoms were photographed under ultraviolet light, and total RNA and total protein from the upper leaves were extracted at the time when viral symptoms became evident in the control. RT‐qPCR and western blot were used to evaluate TMV‐GFP accumulation in each treatment. Each treatment was repeated three times.

Furthermore, we investigated how long the protective effects of CDs@dsCP on plants last. Specifically, subsequent inoculation of TMV‐GFP was conducted at intervals of 1 and 3 days following the application of CDs@dsCP on *N. benthamiana*. In addition, inoculation of TMV‐GFP was also conducted at 5 days following the application of CDs@dsCP on *C. annuum* L. TMV‐GFP accumulation in each treatment group was also evaluated by RT‐qPCR and western blot at 5‐day post inoculation (dpi).

### Extraction of total RNA and RT‐qPCR

Total RNA was extracted from leaves (0.1 g) treated with RN12‐PLANTeasy (Aidlab Biotechnologies Co., Ltd., China). The extracted RNA was quantified using a spectrophotometer (NanoDrop ND‐1000). The RT mix with DNase (US Everbright® Inc., China) was used to reverse transcribe total RNA (800 ng) to cDNA following the vendor's instructions. The mRNA expression level and amount of TMV were determined by quantitative real‐time PCR following the instructions of 2 × SYBR Green qPCR Master Mix (US Everbright® Inc., China) and compared to the expression of *actin7*, which is a housekeeping gene used for normalization of gene expression. Details of the PCR primers used above are also provided in Table S1.

### Western blot

Inoculated leaves (0.1 g) were fully ground after flash freezing in liquid nitrogen and homogenized in plant cell lysis buffer (Beyotime Biotechnology Co., Ltd., China) for 30 min on ice. The mixture was subsequently centrifuged at 12 000 × ***g*** for 10 min at 4 °C, and 20 μL 5× protein loading buffer (Beyotime Biotechnology Co., Ltd., China) was added to the supernatant (80 μL), which was subsequently boiled at 100 °C for 5 min. Protein (7 μL) was loaded onto SDS‐PAGE 10% gradient acrylamide gel (Sangon Biotech Co., Ltd., China), which was subsequently transferred to 0.45 μm polyvinylidene fluoride (PVDF) membranes. The membranes were incubated in 1× TBST (10 mM Tris, 0.15 M NaCl, 0.1% Tween‐20 and pH 7.5) with 5% skim milk powder for 3 h and then transferred to 1× TBST with 5% skim milk powder and a 1:5000 mouse anti‐GFP‐Tag mAb (ABclonal Technology Co., Ltd., China) and incubated overnight at 4 °C. The membranes were treated for 2 h with 1:8000 HRP‐conjugated mouse anti‐rabbit IgG light chain (ABclonal Technology Co., Ltd., China) diluted in 1× TBST with 5% skim milk powder (Beyotime Biotechnology Co., Ltd., China). After washing with 1× TBST, the GFP signal was quantified relative to the total protein signal via the LI‐COR Odyssey Infrared Image Studio software according to the manufacturer's instructions.

### Detection of siRNA

To determine whether the nanomaterial‐delivered dsRNA could generate siRNA in plant cells, we conducted siRNA analysis as follows: Four‐week‐old *N. benthamiana* leaves were treated with CDs@dsCP, nanosheet@dsCP and nanoporous@dsCP complexes via foliar spraying. Leaf samples were collected at 1 and 3 days post‐treatment, following thorough washing with nuclease‐free water to remove surface residues. Total RNA was extracted using TRIzol reagent according to standard protocols.

For siRNA detection, we performed northern blot analysis using the following procedures. First, DNA probes were synthesized using the Biotin Random Prime DNA Labeling Kit (Beyotime Biotechnology Co., Ltd., China), with probe‐specific primers listed in Table S1. Subsequently, siRNA hybridization was carried out following the manufacturer's instructions for the Biotin Northern Blot Kit (for small RNA, Beyotime Biotechnology Co., Ltd., China). A consistent amount of 20 μg total RNA was loaded per lane to ensure comparable detection sensitivity across samples.

### Plant physiological measurements

To clarify whether g‐C_3_N_4_@dsCP treatment affects plant growth and germination rate, the physiological parameters of the plants were measured 10 days after spraying with naked dsCP, CDs, nanosheet, nanoporous, CDs@dsCP, nanosheet@dsCP and nanoporous@dsCP. We measured plant height and root length after different treatments. Fresh samples were immediately weighed and recorded. For dry weight, fresh plants were placed at 60 °C and dried before being weighed and recorded. At least three replicates were performed for each treatment. In addition, we evaluated the effects of the nanomaterials on seed germination. First, we soaked 100 seeds in naked dsCP, CDs, nanosheet, nanoporous, CDs@dsCP, nanosheet@dsCP and nanoporous@dsCP solutions for 12 h each. The seeds were then spread on wetted filter paper, and the seed germination experiments were carried out at room temperature (25 °C). Three replicates were used for each treatment. The seed germination rate was determined, and the seeds were photographed after 4 days.

### Zebrafish and earthworm safety assessment

The acute toxicity of the test materials was evaluated in zebrafish using static bioassays. During the testing period, the fish were not fed. Ten zebrafish were co‐cultured in 20 mg/L aqueous solutions of the following test compounds: dsCP, CDs, nanosheet, nanoporous, CDs@dsCP, nanosheet@dsCP and nanoporous@dsCP, separately. A deionized water group served as the control. After 96 h of exposure, zebrafish survival was recorded, and any deceased individuals were promptly removed to maintain water quality. Meanwhile, the acute toxicity of the compounds to earthworms was assessed using the filter paper contact method, following relevant OECD guidelines. Briefly, filter paper was placed in a 10 cm diameter transparent Petri dish, and 2 mL of each 20 mg/L dsCP, CDs, nanosheet, nanoporous, CDs@dsCP, nanosheet@dsCP and nanoporous@dsCP solution was slowly applied to the paper, separately. Deionized water was used as the control. Ten earthworms were then introduced into each dish, which was subsequently sealed with perforated plastic wrap to allow ventilation. The dishes were incubated at 20 ± 1 °C and 75% relative humidity under dark conditions. After 48 h of exposure, earthworm survival and physiological responses were assessed. Mortality was defined as the absence of movement upon mechanical stimulation. Normal: No observable adverse effects.

### Statistical analysis

Statistical analysis was conducted using SPSS 19.0 software (SPSS Inc.). Independent *t*‐tests were used to determine significant differences between two samples. The other data were analysed using the Tukey HSD test at *P* < 0.05. The descriptive statistics are shown as mean values and standard errors of the mean.

## Supporting information


**Figure S1** High‐resolution X‐ray photoelectron spectra of g‐C_3_N_4_.
**Figure S2** Quantitative fluorescence intensity analysis of agarose gel electrophoresis.
**Figure S3** Laser confocal photographs showing the subcellular localization of three different morphologies of g‐C3N4, including CDs‐Cy3, nanosheet‐Cy3 and nanoporous‐Cy3, in leaves of 16C (*Nicotiana benthamiana* expresses GFP consistently) after they were injected into 16C leaves for 24 h.
**Figure S4** Interference efficiency of different morphologies g‐C3N4@dsGFP on GFP gene expression.
**Figure S5** ABA treatment induces stomatal closure.
**Figure S6** Construction of the prokaryotic expression system and screening of optimal IPTG concentration in prokaryotic cells induced to express dsRNA.
**Figure S7** Northern blot analysis of g‐C_3_N_4_@dsCP derived siRNAs in *N. benthamiana* leaves.
**Figure S8** g‐C_3_N_4_ CDs@dsCP provides long‐lasting antiviral protection to *N. benthamiana*.
**Figure S9** g‐C_3_N_4_ CDs@dsCP provides antiviral protection to *Capsicum annuum* L.
**Figure S10** Safety assessment of g‐C_3_N_4_@dsCP for zebrafishes and earthworms.
**Table S1** All primers used in this study.

## Data Availability

The data that supports the findings of this study are available in the supplementary material of this article. There is no other data (such as omics data) that needs to be stored online in this article.
