# Supplementary material for: Morphology effect of a novel biocompatible nucleic acid delivery nanosystem of g‐C3N4@dsRNA for application in plant gene expression and plant virus disease protection
Source: Plant Biotechnol J. 2025 Jun 18;23(9):3949–66. doi: 10.1111/pbi.70189 (PMC12392969; doi:10.1111/pbi.70189)
Supplement: Supplementary file 1 — Figure S1 High‐resolution X‐ray photoelectron spectra of g‐C3N4. Figure S2 Quantitative fluorescence intensity analysis of agarose gel electrophoresis. Figure S3 Laser confocal photographs showing the subcellular localization of three different morphologies of g‐C3N4, including CDs‐Cy3, nanosheet‐Cy3 and nanoporous‐Cy3, in leaves of 16C (Nicotiana benthamiana expresses GFP consistently) after they were injected into 16C leaves for 24 h. Figure S4 Interference efficiency of different morphologies g‐C3N4@dsGFP on GFP gene expression. Figure S5 ABA treatment induces stomatal closure. Figure S6 Construction of the prokaryotic expression system and screening of optimal IPTG concentration in prokaryotic cells induced to express dsRNA. Figure S7 Northern blot analysis of g‐C3N4@dsCP derived siRNAs in N. benthamiana leaves. Figure S8 g‐C3N4 CDs@dsCP provides long‐lasting antiviral protection to N. benthamiana. Figure S9 g‐C3N4 CDs@dsCP provides antiviral protection to Capsicum annuum L. Figure S10 Safety assessment of g‐C3N4@dsCP for zebrafishes and earthworms. Table S1 All primers used in this study. [file PBI-23-3949-s001.docx]

**Supplemental Materials**

**Figure S1**


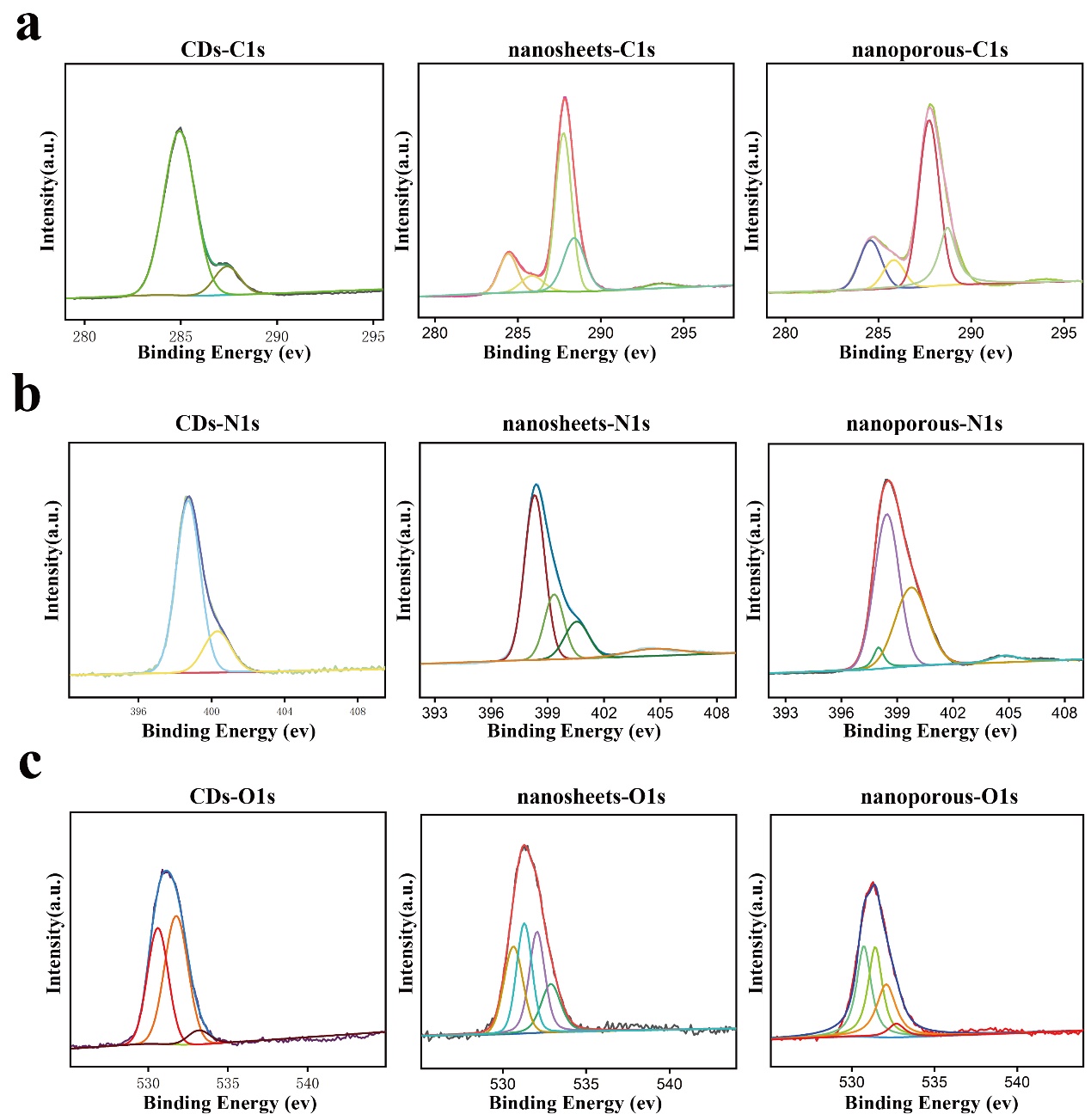


**Figure S1. High-resolution X-ray photoelectron spectra of g-C_3_N_4_.** C1s (a), N1s (b), and O1s(c) spectra of the g-C_3_N_4_ CDs-PEI, g-C_3_N_4_ nanosheet-PEI and g-C_3_N_4_ nanoporous-PEI. All X-ray photoelectron spectra were calibrated using the g-C_3_N_4_ peak at BE = 684.8 eV as the reference.

**Figure S2**


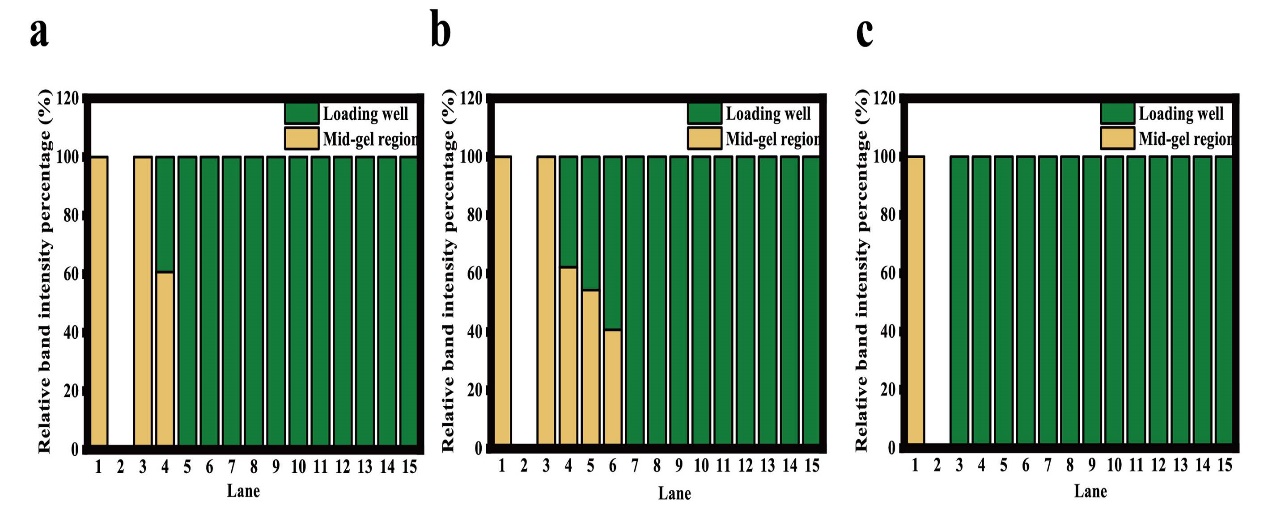


**Figure S2. Quantitative fluorescence intensity analysis of agarose gel electrophoresis.**

The electrophoretic fluorescence intensity percentages of g-C_3_N_4_ and dsGFP were measured at different loading ratios. (a) shows the percentage of fluorescence intensity corresponding to Figure. 1g. (b) shows the percentage of fluorescence intensity corresponding to Figure. 1h. (c) shows the percentage of fluorescence intensity corresponding to Figure. 1i. Fluorescence intensities were quantified using Image Lab software (Bio-Rad) and normalized against the gel matrix background fluorescence.

**Figure S3**


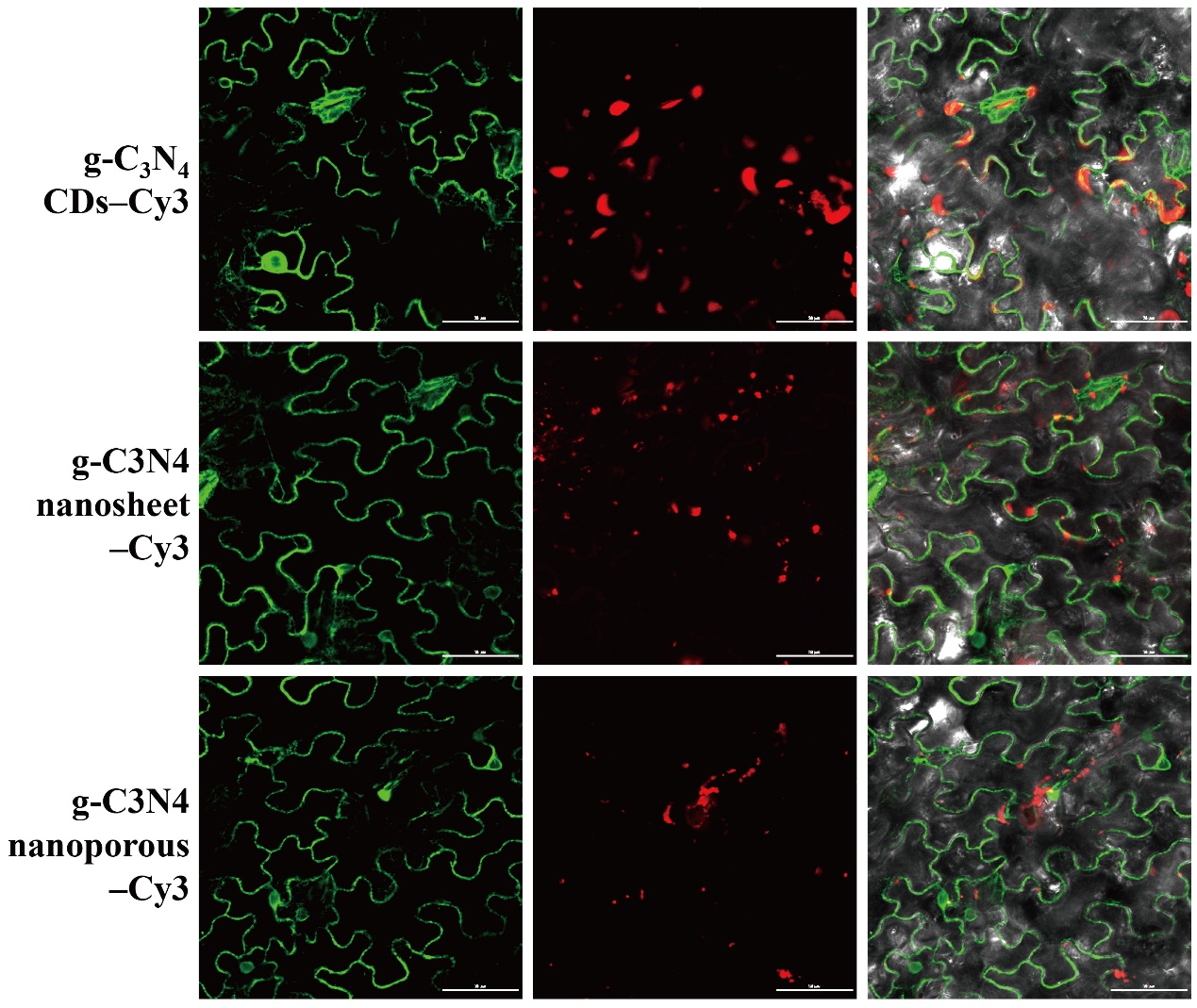


**Figure S3. Laser confocal photographs showing the subcellular localization of three different morphologies of g-C3N4, including CDs-Cy3, nanosheet-Cy3 and nanoporous-Cy3, in leaves of 16C (*Nicotiana* *benthamiana* expresses GFP consistently) after they were injected into 16C leaves for 24h.** The scale bar of all laser confocal is 50 μm.

**Figure S4**


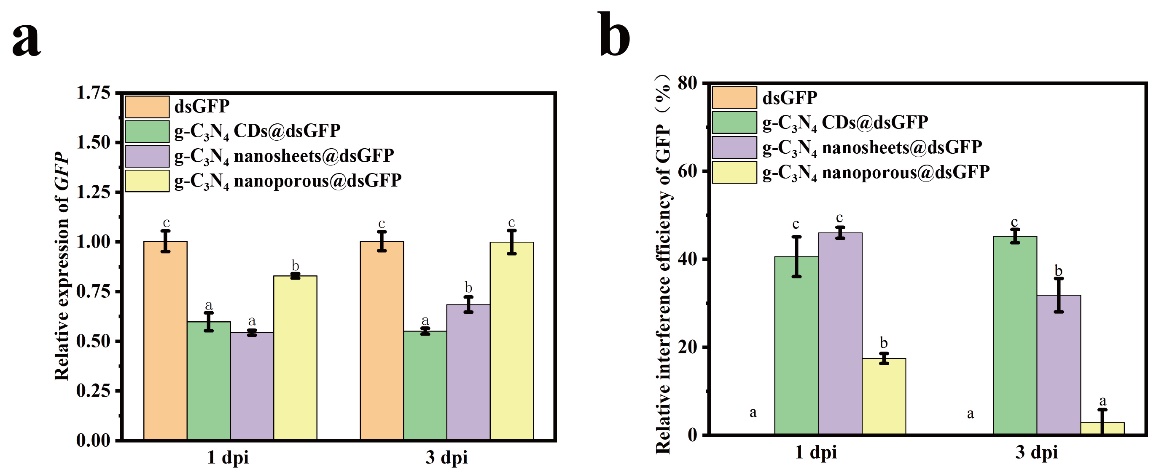


**Figure S4. Interference efficiency of different morphologies g-C_3_N_4_@dsGFP on GFP gene expression.**

(a) The naked dsGFP, g-C_3_N_4_ CDs@dsGFP, g-C_3_N_4_ nanosheet@dsGFP, and g-C_3_N_4_ nanoporous@dsGFP were sprayed on the leaves of 16C. Leaf were sampled at 1 and 3 dps, and the transcript level of the endogenous GFP gene was quantified using RT-qPCR. The treatment of naked dsGFP was employed as the control. Statistical significance was determined at p < 0.05.

(b) GFP silencing efficiency was assessed by comparing the treatment groups (g-C_3_N_4_ CDs@dsGFP, g-C_3_N_4_ nanosheet@dsGFP, and g-C_3_N_4_ nanoporous@dsGFP) to the control group (naked dsGFP). The relative silencing efficiency was calculated using the following formula:

$$Relative silencing efficiency (\%)=\frac{control group-treatment group}{control group}x100$$

**Figure S5**


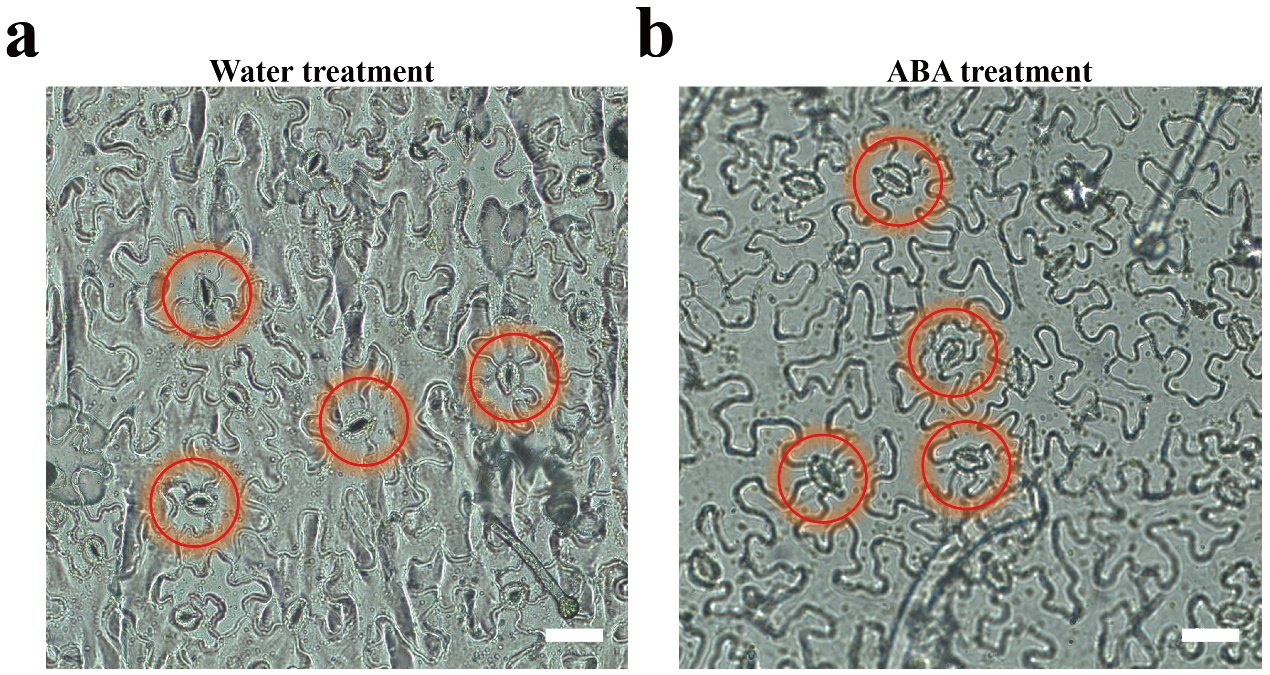


**Figure S5.** **ABA treatment induces stomatal closure.** Leaves were evenly sprayed with 100 mmol/L ABA solution. Stomatal opening and closure in N. benthamiana leaves were observed after 4 hours using a Zeiss microscope. (a) Pour plenty of water over the roots resulted in stomata remaining open. (b) ABA treatment induced stomatal closure.

**Figure S6**


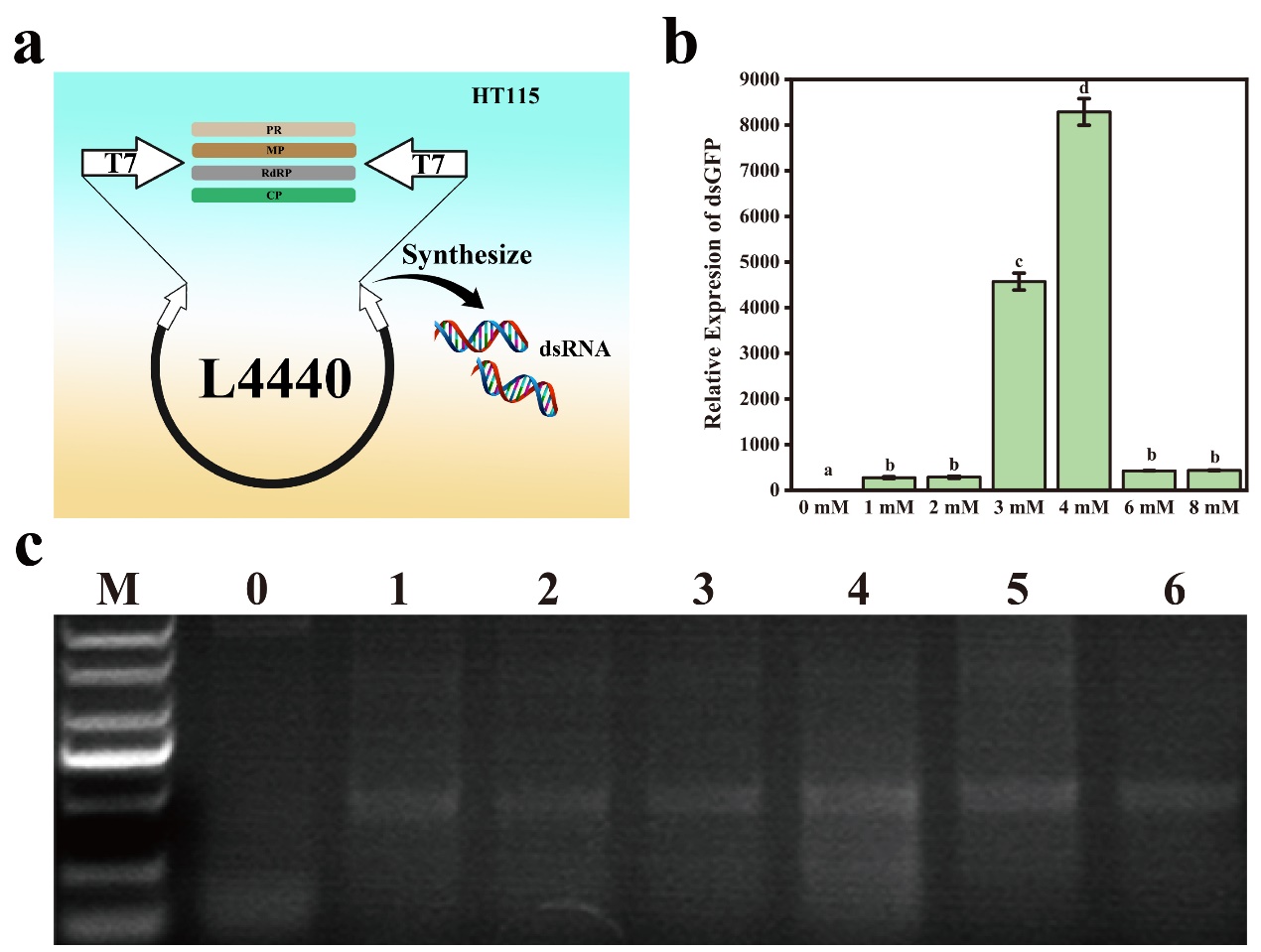


**Figure S6. Construction of the prokaryotic expression system and screening of optimal IPTG concentration in prokaryotic cells induced to express dsRNA.**

(a) Schematic diagram of the recombinant vector construction and dsRNA preparation. The biosynthesis of dsRNA corresponding to partial sequences of the TMV target genes (RdRP, PR, MP, and CP) was carried out in E. coli HT115 (DE3).

(b) RT-qPCR analysis of the relative expression of dsGFP. dsGFP synthesis was induced with different IPTG concentrations (0 mM, 1 mM, 2 mM, 3 mM, 4 mM, 6 mM, 8 mM).

(c) Gel electrophoresis image showing the biosynthesis of dsGFP in E. coli HT115 (DE3) under different IPTG induction concentrations.

**Figure S7**


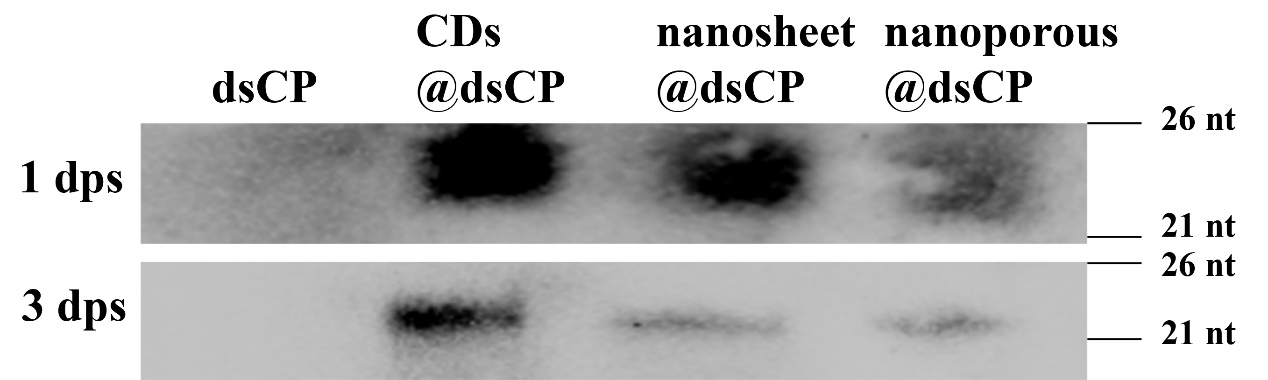


**Revised Figure S7. Northern blot analysis of g-C_3_N_4_@dsCP derived siRNAs in *N. benthamiana* leaves.** Total RNA was extracted from leaves at 1 and 3 dps with: (1) naked dsCP, (2) CDs@dsCP, (3) nanosheet@dsCP, and (4) nanoporous@dsCP, after thorough washing with nuclease-free water to remove uninternalized dsCP. RNA samples (20 μg per lane) were separated by 15% urea-PAGE, transferred to nylon membranes, and hybridized with biotin-labeled CP probes.

**Figure S8**


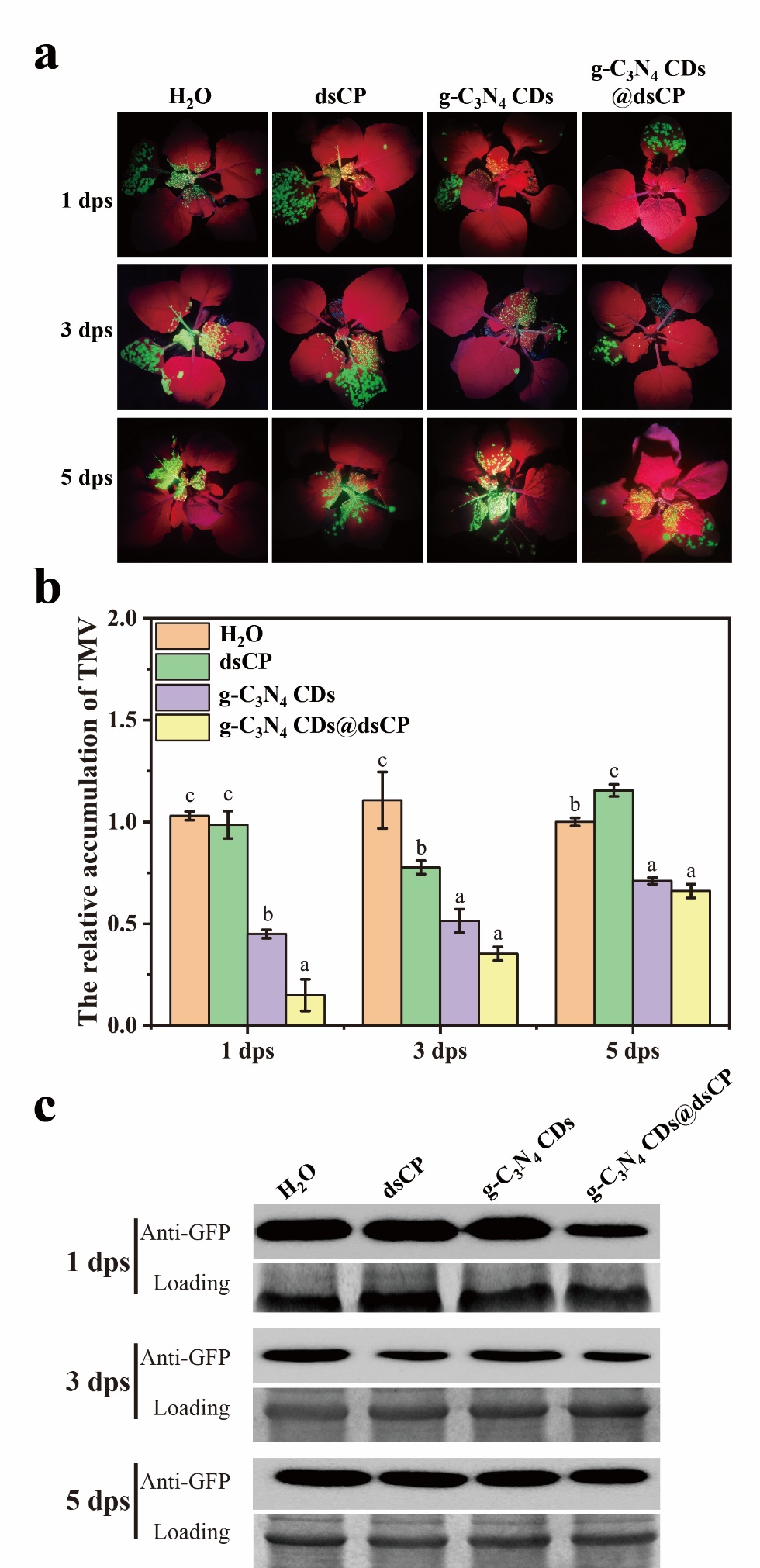


**Figure S8. g-C_3_N_4_ CDs@dsCP provides long-lasting antiviral protection to *N*. *benthamiana*.**

(a) Phenotypes of TMV-GFP infection in N. benthamiana. Leaves were treated with naked dsCP, g-C_3_N_4_ CDs, or g-C_3_N_4_-CDs@dsCP, followed by inoculation with TMV-GFP at 1 dpi, 3 dpi, and 5 dpi. Each treatment was performed with at least three biological replicates.

(b) The accumulation of TMV-GFP for each sample was quantified by RT-qPCR. Different letters above each bar indicate significant differences at *p* < 0.05 as determined by one-way ANOVA with Tukey’s HSD multiple comparison post hoc test. Bars represent the mean ± SE.

(c) Western blot detection of the GFP protein content of TMV-GFP for different treated plants at 7 dpi. Loading stands for Coomassie bright blue staining. All experiments were repeated at least three times with similar results.

**Figure S9**


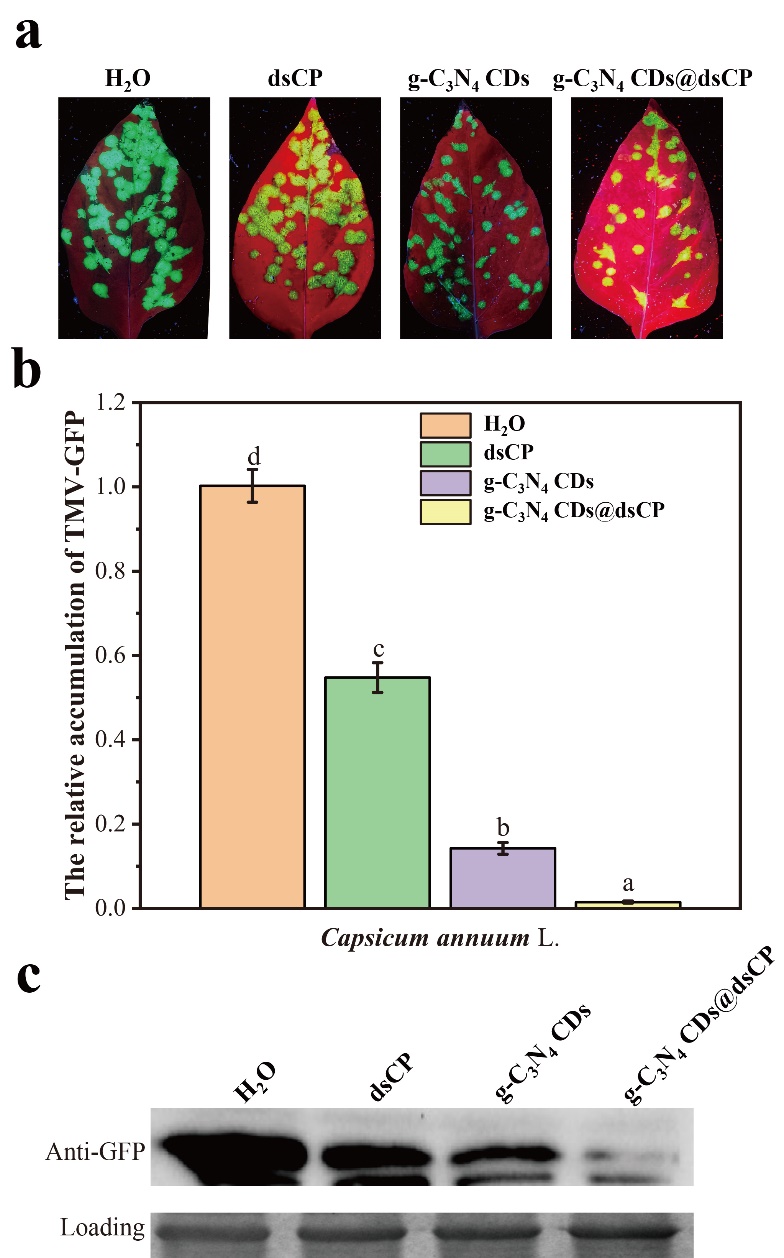


**Figure S9. g-C_3_N_4_ CDs@dsCP provides antiviral protection to *Capsicum* *annuum* L..**

(a) Phenotypes of TMV-GFP infection in pepper plants. Leaves were treated with bare dsCP, g-C_3_N_4_ CDs, or g-C_3_N_4_@dsCP, followed by inoculation with TMV-GFP at 5 dps. Photographs were taken at 7 dpi. Each treatment was performed with at least three biological replicates.

(b) The accumulation of TMV-GFP for each sample was quantified by RT-qPCR. Different letters above each bar indicate significant differences at *p* < 0.05 as determined by one-way ANOVA with Tukey’s HSD multiple comparison post hoc test. Bars represent the mean ± SE.

(c) Western blot detection of the GFP protein content of TMV-GFP for different treated plants at 7 dpi. Loading stands for Coomassie bright blue staining. All experiments were repeated at least three times with similar results.

**Figure S10**


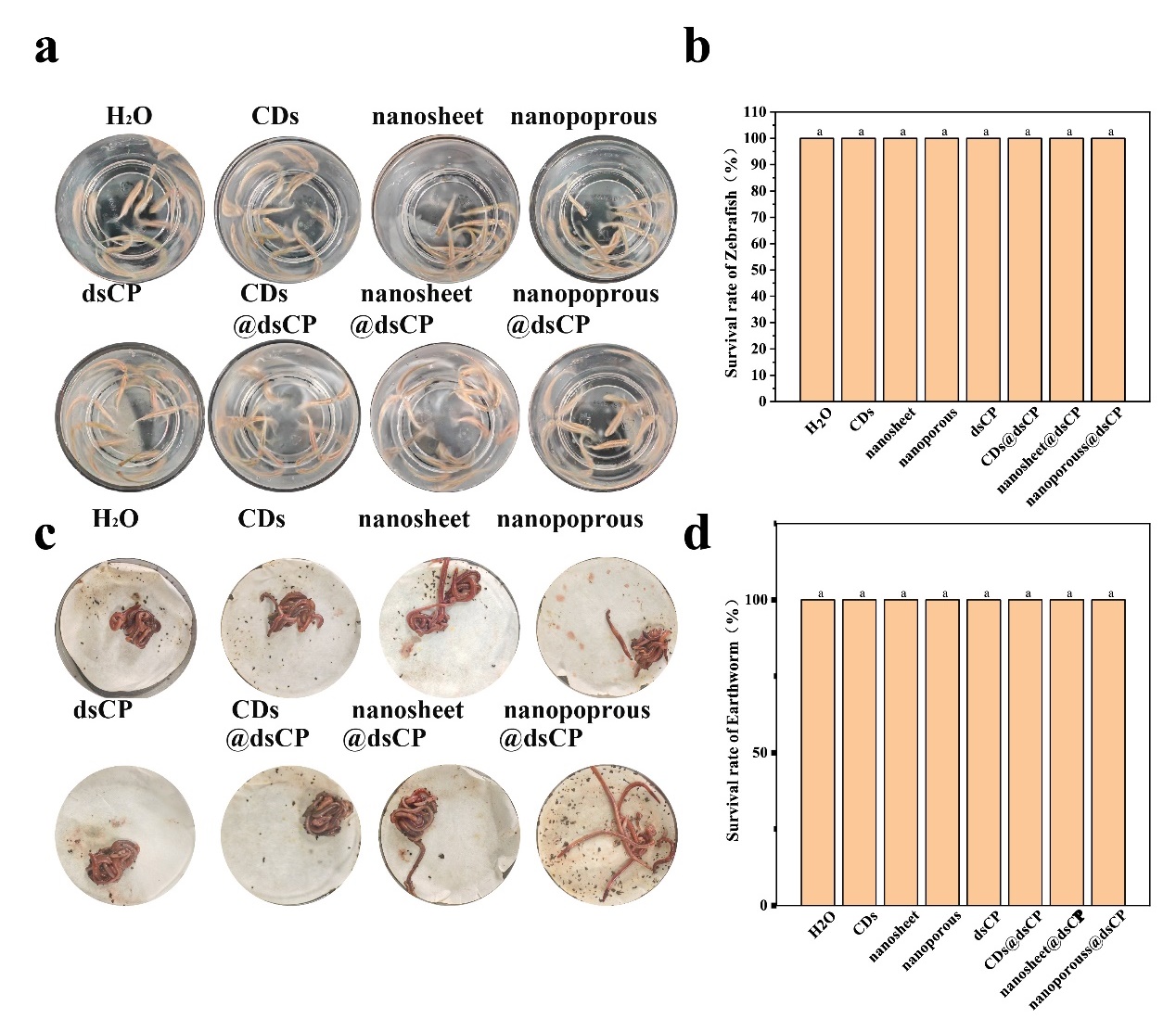


**Figure S10 Safety assessment of g-C_3_N_4_@dsCP for zebrafishes and earthworms.**

(a) Photographs of zebrafishes in an acute toxicity test after exposure to dsCP, CDs, CDs@dsCP, nanosheet, nanosheet@dsCP, nanoporous, or nanoporous@dsCP at 20 μg/mL for 96 h. (b) Survival rate of zebrafish. (c) Photographs of earthworms in an acute toxicity test after exposure to dsCP, CDs, CDs@dsCP, nanosheet, nanosheet@dsCP, nanoporous, or nanoporous@dsCP at 20 μg/mL for 48 h. (d) Survival rate of earthworms.

**Supplemental Table 1. All primers used in this study**

| **Primers name** | **Squence (5' to 3')** | **Experiments** |
| --- | --- | --- |
| **L4440-CP-F** | **TCATCGATGAATTCGAGCTCCCTTATACAATCAACTCTCCGA** | **Obtaining the dsRNA sequence of dsCP from TMV** |
| **L4440-CP-R** | **CTTGATATCGAATTCCTGCAGGAACCAGTTCATTAGCCAAA** |  |
| **L440-RdRP-F** | **TGATATCATCGATGAATTCGAGCTCTGGCATACACACAGACAGCTAC** | **Obtaining the dsRNA sequence of dsRdRP from TMV** |
| **L4440-RdRP-R** | **AAGCTTGATATCGAATTCCTGCAGCTCGAACGTCCAGGTTGGGCA** |  |
| **L4440-MP-F** | **GATATCATCGATGAATTCGAGCTCTGGCTCTAGTTGTTAAAGGA** | **Obtaining the dsRNA sequence of dsMP from TMV** |
| **L44440-MP-R** | **AAGCTTGATATCGAATTCCTGCAGATCGCGTCCTGGGTGGTTAT** |  |
| **L4440-RP-F** | **GATATCATCGATGAATTCGAGCTCGCCACGCCAGACTGGACTATTG** | **Obtaining the dsRNA sequence of dsPR from TMV** |
| **L4440-RP-R** | **AAGCTTGATATCGAATTCCTGCAGCAACGGGCCGAATATTGCATTG** |  |
| **L4440-GFP-F** | **GATATCATCGATGAATTCGGAGAAGAACTTTTCACTGGAG** | **Obtaining the dsRNA sequence of dsGFP from 16C** |
| **L4440-GFP-R** | **AAGCTTGATATCGAATTCGAGGATGTTTCCGTCCTCCTTG** |  |
| **L4440-T7-F** | **GAGCGCAGCGAGTCAGTGAG** | **For in vitro transcription** |
| **L4440-T7-R** | **TGCAAGGCGATTAAGTTGGG** |  |
| **qPCR-GFP-1F** | **GCGATGGCCCTGTCCTTTTA** | **Detection of GFP expression in 16C** |
| **qPCR-GFP-1R** | **TGCCATGTGTAATCCCAGCA** |  |
| **qPCR-GFP-2F** | **ACGTGCTGAAGTCAAGTTTG** | **Examining the stability of dsRNA *in* *vitro*** |
| **qPCR-GFP-2R** | **ACGTGTCTTGTAGTTCCCGT** |  |
| **qPCR-TMV-GFP-F** | **TCCATGGCCAACACTTGTCA** | **Detection of TMV-GFP accumulation** |
| **qPCR-TMV-GFP-R** | **TCAGCACGCGTCTTGTAGTT** |  |
| **qPCR-actin-F** | **CTTGAAACAGCAAAGACCAGC** | **The reference gene in *N*. *benthamiana*** |
| **qPCR-actin-R** | **CATCCTATCAGCAATGCCCG** |  |
| **qPCR-GapA-F** | **ATATGCTGGCCAGGACATCG** | **The reference gene in *E*. *coli*** |
| **qPCR-GapA-R** | **CGTGAACGGTGGTCATCAGA** |  |
| **Biotin-TMV CP-F** | **GCAACAAGCTAGGACAACAGTC** | **Biotin-labeled probes were synthesized** |
| **Biotin-TMV CP-R** | **TGAACATGCCAGTTCCACGA** |  |
